# Laminin γ3 plays an important role in retinal lamination, photoreceptor organisation and ganglion cell differentiation

**DOI:** 10.1038/s41419-018-0648-0

**Published:** 2018-05-23

**Authors:** Birthe Dorgau, Majed Felemban, Alexander Sharpe, Roman Bauer, Dean Hallam, David H. Steel, Susan Lindsay, Carla Mellough, Majlinda Lako

**Affiliations:** 1 Institute of Genetic Medicine, Newcastle University, Newcastle upon Tyne, United Kingdom; 2 Institute of Neuroscience, Newcastle University, Newcastle upon Tyne, United Kingdom; 3 Lions Eye Institute, Centre for Ophthalmology and Visual Science, University of Western Australia, Nedlands WA, Australia

## Abstract

Laminins are heterotrimeric glycoproteins of the extracellular matrix. Eleven different laminin chains have been identified in vertebrates. They are ubiquitously expressed in the human body, with a distinct tissue distribution. Laminin expression in neural retina and their functional role during human retinogenesis is still unknown. This study investigated the laminin expression in human developing and adult retina, showing laminin α1, α5, β1, β2 and γ1 to be predominantly expressed in Bruch’s membrane and the inner limiting membrane. Laminin-332 and laminin γ3 expression were mainly observed in the neural retina during retinal histogenesis. These expression patterns were largely conserved in pluripotent stem cell-derived retinal organoids. Blocking of laminin γ3 function in retinal organoids resulted in the disruption of laminar organisation and synapse formation, the loss of photoreceptor organisation and retinal ganglion cells. Our data demonstrate a unique temporal and spatial expression for laminins and reveal a novel role for laminin γ3 during human retinogenesis.

## Introduction

The extracellular matrix (ECM) is a non-cellular structure that is present in all tissues and can be divided into two separate types, the connective tissue matrix and the basement membrane^1^. The ECM provides structural support and promotes cellular functions including differentiation, adhesion, migration, proliferation, axonal growth and morphogenesis in many tissues^1–4^. Laminins (Lam) are a family of heterotrimeric glycoproteins and part of the ECM^5^. They are a major component of basement membranes and are important for multiple biological processes^4,5^. Laminins are composed of one α, one β and one γ chain, and at present 11 different laminin chains have been identified in vertebrates: five α, three β and three γ chains^6^. These 11 laminin chains can assemble into at least 16 different laminin isoforms, named according to their trimer composition^7^. All isoforms have a tissue-specific distribution^6^. Mutations in different laminin chains are known to cause several congenital diseases in human including eye abnormalities^8–13^.

Laminin functions have often been associated with embryogenesis. For example, laminin-111 has been shown to play an essential role in primitive ectoderm differentiation^14–16^ and knockdowns of any individual chain of laminin-111 result in lethality^15,17,18^. Laminin-111 and other laminin chains play an important role during optic cup morphogenesis and retinal histogenesis^19–21^, photoreceptor structure and synapse formation^20,22,23^, stability of the inner limiting membrane (ILM)^24^ and retinal ganglion cell (RGC) axonal growth^25–29^.

Only two studies to date have investigated the temporal and spatial distribution pattern of laminin during retinogenesis in rat and human^30,31^, showing that laminin expression varies between developmental stages and species, however, the laminin distribution in developing human neural retina has not been systematically studied.

Human pluripotent stem cells (hPSCs), including human embryonic stem cells (hESCs) and human induced pluripotent stem cells (hiPSCs), offer opportunities for research in human embryogenesis, disease modelling and cell replacement therapies. Several reports have shown the successful generation of self-organising retinal organoids from mouse embryonic stem cells and hPSCs under three-dimensional (3D) culture conditions^32–38^. These retinal organoids recapitulate many aspects of in vivo retinogenesis^19,39,40^ and comprise key retinal cell types, which respond to light and electrophysiological stimuli^41–44^.

In this study, we set out to investigate the temporal and spatial distribution of most laminin chains in different species and, importantly, during human retinal development and hPSC-derived retinal organoids. Our data show a distinct temporal and spatial expression pattern for each laminin during human retinogenesis and reveal an important role for Lamγ3 in retinal lamination, organisation of the ONL and differentiation of RGCs in hPSC-derived retinal organoids.

## Materials and methods

### Tissue preparation for Immunohistochemistry

In this study mouse, macaque and human eye tissue was prepared for experiments following identical protocols. Adult female and male wild-type mice (C57BL/6) obtained through the Functional Genetic Unit (Institute of Genetic Medicine) at Newcastle University were sacrificed by cervical dislocation and the eyes enucleated. Adult macaque eyes were obtained from Newcastle University following guidelines approved by the ethics committee at Newcastle University and carried out in accordance with the guidelines of the UK Home Office, under control of the Animals (Scientific Procedures) Act 1986. Post mortem adult human eyes donated from one 71- and one 86-year-old male were obtained through Manchester Eye Bank, Manchester Royal Eye Hospital. Human embryonic/foetal eyes aged between 6.3 and 18 post-conception weeks (PCW) were obtained from the MRC/Wellcome Trust funded Human Developmental Biology Resource at Newcastle University (HBDR), with appropriate maternal written consent and approval from the Newcastle and North Tyneside NHS Health Authority Joint Ethics Committee. The HDBR is regulated by the UK Human Tissue Authority (HTA) and operates in accordance with the relevant HTA Codes of Practice.

The cornea and lens were removed from all eyes and the remaining posterior eyecups were fixed in 4% paraformaldehyde (PFA; Santa Cruz Biotechnologies) for 20 minutes–12 hours depending on the tissue size. After several washing steps in physiological phosphate-buffered saline (PBS; pH 7.4), eyecups were cryoprotected in PBS containing 30% sucrose overnight at 4 °C and embedded in OCT (Optimal Cutting Temperature) compound(Cell Path Ltd, Newtown, UK). Sagital sections were cut on a cryostat (Leica Cm1860). The thickness of the section was dependent on the tissue: 20 µm for mouse and monkey eyes, 30 µm for adult human eyes and 10–12 µm for human embryonic/foetal eyes.

### hPSC culture and retinal organoid differentiation

Retinal organoids were generated as described previously with some modifications^37^. Briefly, hESCs (H9; WiCell Inc.) and hiPSCs derived from adult fibroblast (SB-Ad3^45^) were expanded in mTESR™1 (StemCell Technologies, 05850) on growth factor-reduced Matrigel (BD Biosciences, San Jose, CA) coated plates at 37 °C and 5% CO_2_. In contrast to the protocol described by Mellough et al.^37^, the medium was supplemented with 10 µM Rock inhibitor (Y-27632 dihydrochloride; Chemdea, Ridgewood, USA) for the first 2 days of differentiation and with 10% foetal calf serum (FCS; Life Technologies, UK), T_3_ (40 ng/ml; Sigma-Aldrich, UK), Taurine (0.1 mM; Sigma-Aldrich) and retinoic acid (0.5 µM; Sigma-Adrich) after day 18 of differentiation. Retinal organoids were collected at days 35, 90, 150 and 200 of differentiation and processed for immunohistochemistry (IHC) by fixation in 4% PFA for 20–30 minutes followed by several washing steps in PBS. After cryoprotection in PBS containing 30% sucrose overnight, organoids were embedded in OCT (Cell Path Ltd, Newtown, UK) and sections (10 µm) were cut on a cryostat (Leica Cm1860).

### Immunohistochemistry

Sections were rinsed in PBS and incubated for 1 hour at room temperature in blocking solution (10% normal goat serum, 0.3% Triton-X-100 in PBS). All antibodies were diluted in antibody diluent solution [ADS; 1% bovine serum albumin (BSA), 0.3% Triton-X-100 in PBS]. Primary antibodies (Table S1) were applied overnight at 4 °C. After several washing steps in ADS, section were incubated with secondary goat antibodies conjugated either to Alexa488 (Life Technologies), Cy3 (Jackson Immuno Research Laboratories) or Alex647 (Life Technologies) for 2 hours at room temperature in the dark. Then sections were washed several times in PBS and mounted with VectaShield (Vector Laboratories, Burlingame, CA) containing Hoechst (Life Technologies). For each antibody, control IHC was carried out by omitting the primary antibody.

### Laminin blocking experiments

Blocking of Lamγ3 was performed at days 43 and 150 of retinal organoid differentiation. For blocking of Lamγ3, the medium was supplemented with a rabbit IgG anti-laminin γ3 antibody (10 µg/ml; Table S1). Medium was changed every 2–3 days and the experiment was stopped after 7 days. Brightfield images of retinal organoids were taken after every media change using a Zeiss AxioVert1 (Zeiss, Germany) with a 5 × /0.15 air objective. After experiments, retinal organoids were fixed and further processed for IHC as described above, or rinsed in PBS and immediately frozen at −80 °C for quantitative reverse transcriptase-PCR (qRT-PCR). For both experiments, two individual experimental replicates were carried out for IHC and three for qRT-PCR. Quantification of Caspase-3 was performed with ImageJ (NIH, Bethesda, MD). The quantitative data included in the results section represent the mean ( ± SEM) of Caspase-3 expression in control and group treated with Lamγ3 antibody.

### Image acquisition and analysis

Images were taken using a Zeiss Axio ImagerZ2 equipped with an Apotome.2 and Zen 2012 blue software (Zeiss, Germany). Scanning of image stacks was performed with either a 20 × /0.8 air objective or a 40 × /1.3 oil immersion objective using a *z-*axis increment of either 0.49 µm for 20 × air objective or 0.28 µm for 40 × oil immersion objective. Two independent replicates were obtained for adult mouse, monkey, human and embryonic eye tissues, as well as for retinal organoids. In all, 10 to 15 retinal organoids were collected per replicate and 3–5 examples were imaged at each time point at which a representative image is shown here. Final images are presented as a maximum projection and adjusted for brightness and contrast in Adobe Photoshop CS6 (Adobe Systems).

### qRT-PCR analysis

In total, 15-20 retinal organoids were homogenised using a Dounce Tissue Grinder (Sigma-Aldrich, UK) and RNA was extracted using the Promega tissue extraction kit (Promega, USA) as per manufacturer’s instructions. In all, 1 μg of RNA was reverse transcribed using random primers (Promega, USA). qRT-PCR was performed using a Quant Studio 7 Flex system (Applied Biosystems, USA) with SYBR Green (Promega, USA). Each primer (Table S2) was used at a concentration of 1 μM, and at a ratio of 50:50 for forward and reverse. The reaction parameters were as follows: 95 °C for 15 minutes to denature the complementary DNA and primers, 40 cycles of 94 °C for 15 seconds followed by primer specific annealing temperature for 30 seconds, succeeded by a melt curve. The data were analysed using 2-ΔΔCt method. Statistical analysis was done using Prism 6 (GraphPad Software, La Jolla, CA). All results were validated using Student’s *t*-test for paired samples.

## Results

### Distribution of laminins during retinal development and in adult retina

We examined the distribution of all existing laminin chains in different adult species, human developing retina, as well as hPSC-derived retinal organoids using IHC. Laminin α2 has no retinal-associated expression (except with the retinal vasculature), nor is implicated in any eye-related disease^30,31^, for this reason it was excluded from this study.

#### Laminin expression in the adult mouse, macaque and human retina

**Fig. 1:**
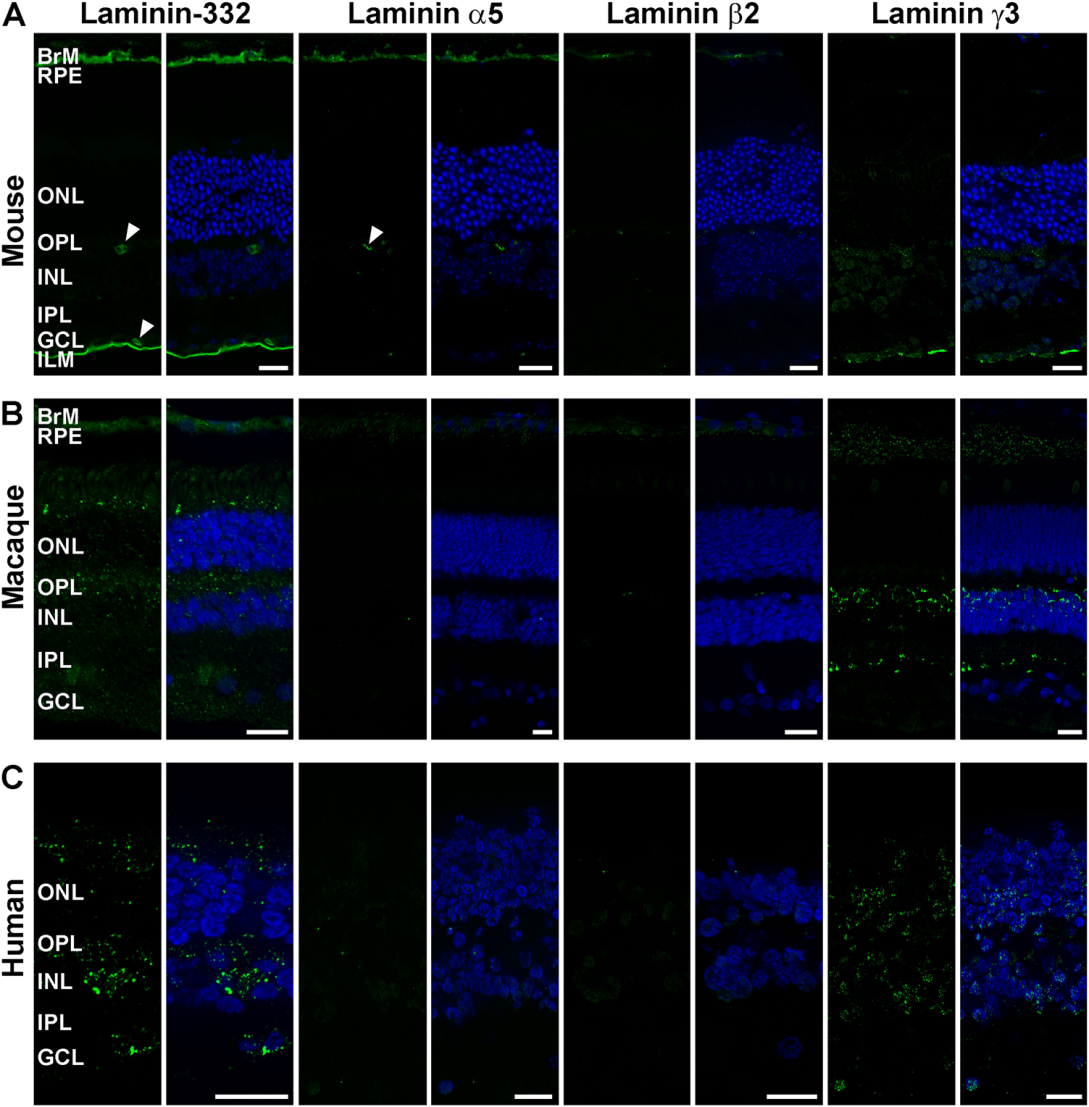
Expression of laminin-332, laminin α5, laminin β2 and laminin γ3 in adult mouse, macaque and human retina. Laminin-332 (green) is expressed in BrM and the ILM of the mouse retina (**a**), the IPM, OPL and GCL of the macaque retina (**b**) and in ONL, INL and GCL of human retina (**c**). Laminin α5 (green) and laminin β2 (green) were found in BrM of mouse retina (**a**) and not in macaque (**b**) and human retina (**c**). Laminin γ3 (green) is expressed in INL and GCL of mouse retina (**a**), in IPM, INL and IPL of macaque retina (**b**) and in ONL, INL and GCL of human retina (**c**). Nuclei are counterstained with Hoechst (blue). Arrowheads indicate expression of laminin-332 and laminin α5 in blood vessels in the mouse retina (**a**). *BrM* Bruch’s membrane, *RPE* retinal pigment epithelium, *IPM* interphotoreceptor matrix, *ONL* outer nuclear layer, *OPL* outer plexiform layer, *INL* inner nuclear layer, *IPL* inner plexiform layer, *GCL* ganglion cell layer, *ILM* inner limiting membrane. Scale bars, 20 μm

A polyclonal antibody that recognises all three chains of laminin-332 showed diverse expression pattern in all three species (Fig. 1). Strong immunoreactivity was found in Bruch's membrane  (BrM) and ILM of the mouse retina (Fig. 1a), whereas laminin-332 was expressed throughout the macaque and human retina (Figs. 1b, c). These findings are consistent with expression data previously obtained in human retina^31^. In all species, no immunoreactivity was found for Lamα1 (Figure S1), corroborating previous data obtained in adult rat and human retina^31^. Lamα4 is expressed in mouse and macaque BrM, as well as in the ILM of the macaque retina (Figure S1A). This laminin chain is, however, absent in the human retina (Figure S1C). Expression of Lamα5, β1 and β2 was found in BrM of mouse retina and no immunoreactivity was seen in the mature macaque and human retina (Fig. 1, S1). Lamγ1 expression was observed in BrM of mouse and macaque retina (Figure S1A, B) and human ILM (Figure S1C). Like laminin-332, Lamγ3 expression varied across all three species (Fig. 1). In the mouse retina, weak immunostaining was found in the inner nuclear layer (INL) and the ganglion cell layer (GCL; Fig. 1a) whereas prominent staining was seen in the macaque interphotoreceptor matrix (IPM; close to the retinal pigment epithelium (RPE)), INL and inner plexiform layer (IPL; Fig. 1b). Higher magnification of Lamγ3 immunostaining in the INL (data not shown) suggests its expression in a specific bipolar cell (BC) type stratifying their axon terminals in the innermost ON layer of the IPL close to the GCL, indicating most likely an ON BC type or Rod BC. However, in human retina Lamγ3 revealed a punctate pattern across all three nuclear layers (Fig. 1c).

Laminin-332, Lamβ2, Lamα5 in the mouse retina (arrowheads; Fig. 1a), as well as Lamγ1 expression in the human retina (arrowhead; Figure S1C) was also associated with retinal vasculature. All results are summarised in Table S3.

#### Distribution of laminins in human embryonic/foetal retina

**Fig. 2:**
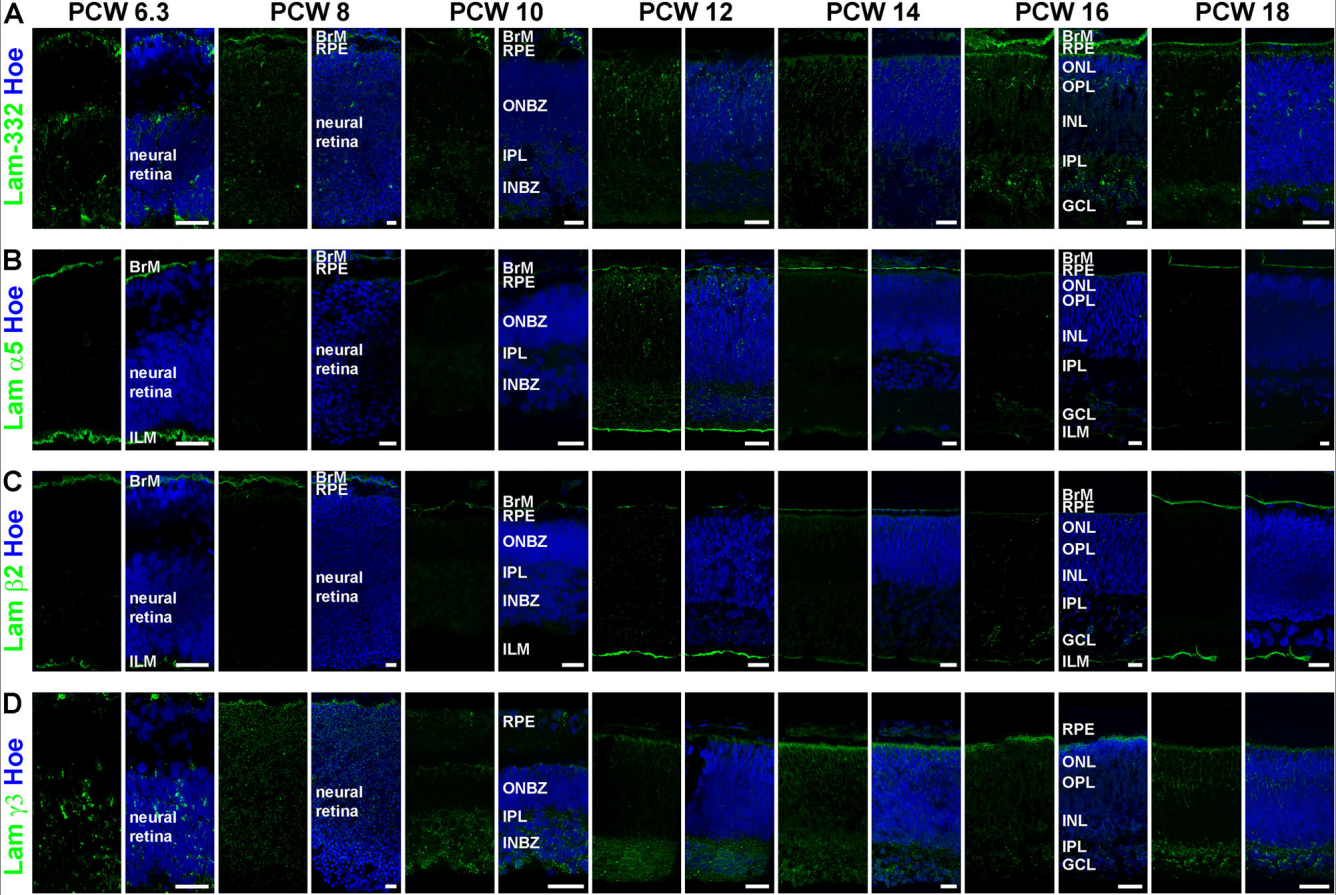
Expression of laminin-332, laminin α5, laminin β2 and laminin γ3 in developing human retina. **a)** Laminin-332 (green) is expressed in BrM and throughout the retina in all developmental stages. **b)** Laminin α5 (green) was found in BrM and the ILM throughout development and additionally in ONBZ, INBZ and IPL at 12 PCW. **c**) Laminin β2 (green) was observed in BrM developmental stages as well as in INBZ at 16 PCW. **d**) Laminin γ3 (green) is expressed throughout the retina during development. Nuclei are counterstained with Hoechst (blue). BrM Bruch’s membrane, RPE retinal pigment epithelium, ONBZ outer neuroblastic zone, ONL outer nuclear layer, OPL outer plexiform layer, INBZ inner neuroblastic zone, INL inner nuclear layer, IPL inner plexiform layer, GCL ganglion cell layer, ILM inner limiting membrane, Hoe Hoechst, Lam laminin, PCW post conceptual week. Scale bars, 20 μm

Laminin chain expression in the human retina was examined at several developmental stages from 6.3 to 18 PCW. Laminin-332 was found at all developmental stages studied, revealing expression in BrM and a punctate pattern throughout the neural retina, except for the 10^th^ PCW where expression was observed only in the inner neuroblastic zone (INBZ; Fig. 2a). Later in development (12–18 PCW), laminin-332 immunoreactivity was also observed in the outer neuroblastic zone (ONBZ) and the developing IPM (Fig. 2a). Lamα1 was expressed in BrM and the ILM at 6.3 PCW, was absent at 8 PCW and was found again at 10 PCW, but only in BrM (Figure S2A). Starting from the 12^th^ PCW onwards, Lamα1 immunoreactivity was restricted to the ILM, except for the 14^th^ PCW (Figure S2A). Immunoreactivity for Lamα4 was first observed during the 12^th^ PCW, showing expression in BrM and IPM (Figure S2B). At 14 PCW, Lamα4 expression was observed in the IPM and ONBZ; however, no expression was detected at 16 and 18 PCW (Figure S2B). Lamα5 and β2 were found in BrM and the ILM throughout all developmental stages (Figs. 2b, c), which is consistent with previously reported data in human foetal retina^30^. In addition, a punctate immunostaining pattern was observed across the entire retina at 12 PCW for Lamα5 (Fig. 2b) and in the INBZ at 16 PCW for Lamβ2 (Fig. 2c). Although the Lamβ1 expression showed some discrepancies, the expression was generally found in BrM and the ILM from 12 PCW onwards (Figure S2C), corroborating data obtained by Byström et al^30^.

Immunoreactivity for Lamγ1 was found throughout the retina, including the IPM, and the ILM at 6.3 PCW. This expression changed from 8 PCW onwards, revealing Lamγ1 in BrM in subsequent developmental stages, except for 10 PCW (Figure S2D). Additionally, Lamγ1 was observed in the ILM at 10, 12, 16 and 18 PCW (Figure S2D), suggesting maintained expression from the 10^th^ PCW onwards as previously reported^30^. Lamγ3 was widely expressed in the retina at all developmental stages studied (Fig. 2d). A punctate pattern throughout the retina was seen at 6.3 and 8 PCW. In the 10th and 12^th^ PCW samples, Lamγ3 expression was seen in the IPM, IPL and INBZ, whereas Lamγ3 immunoreactivity was additionally found in the ONBZ (at 14 and 16 PCW) and later in development (18 PCW) in the outer nuclear layer (ONL; Fig. 2d). All results are summarised in Table S4.

To confirm these findings and investigate the dynamics of laminin chain expression at transcriptional level, we took advantage of a recent RNA-seq study performed by our group^46^ in human developing retina from 4.6 to 18 PCW. This study defined three transcriptional windows: 4.6–7.2 PCW, 7.7–10 PCW and 12–18 PCW, which follow in vivo retinogenesis and correspond to the emergence of optic cup/RPE/lens, RGCs, and photoreceptors/interneurons/Müller cells (MCs), respectively. Analysis of gene expression showed a significant downregulation of Lamα1, α3, α 4, β1, β2 and γ1 from the first (4.6–7.2 PCW) to the second developmental window (7.7–10 PCW), suggesting an important role for these laminin chains in the very early event associated with optic cup, RPE and lens emergence (Figure S3A-C,E,F,H). Notwithstanding this, a few laminin genes namely Lamα1, α3 and β1 showed a significant upregulation from the second to the third developmental window (12–18 PCW), which highlights their potential involvement in retinal cell differentiation (Figure S3A,B,E). In contrast, Lamγ1-3 were significantly downregulated from the second to the third developmental window (Figure S3H-J), which may be due to their restricted expression pattern in certain areas of the developing retina as shown by IHC (Fig. 2d, S2D).

#### Expression of laminins in retinal organoids derived from hESC and hiPSC

**Fig. 3:**
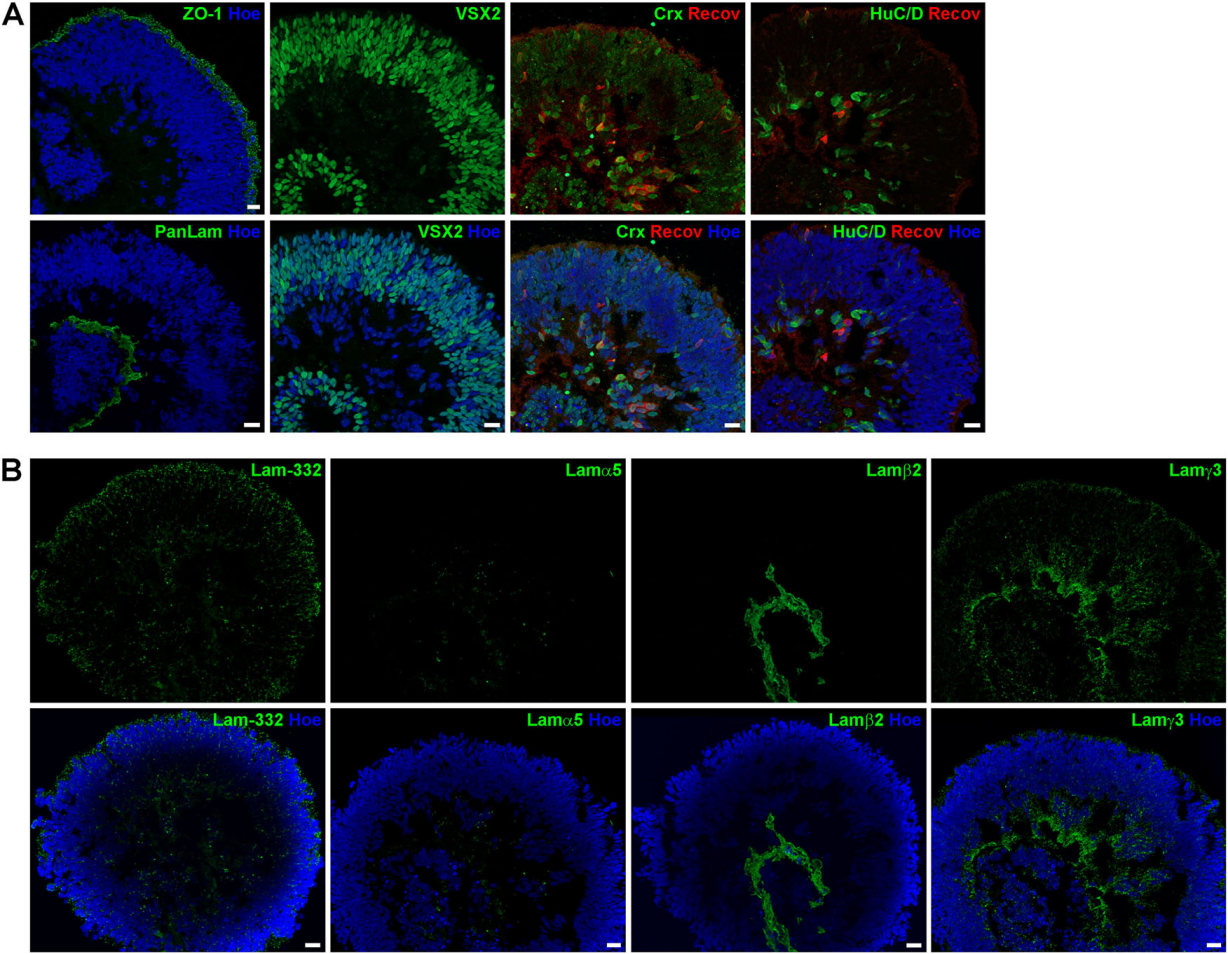
Expression of retinal markers and laminin-332, laminin α5, laminin β2 and laminin γ3 in retinal organoids derived from hESCs at day 35 of differentiation. **a)** Expression of ZO-1 (green), Pan-Laminin (PanLam, green), retinal progenitor cells (VSX2, green), photoreceptors (CRX, green; Recoverin, red) and ganglion cells (HuC/D, green). **b)** Laminin-332 (green) is expressed throughout the retinal organoid. No immunoreactivity for laminin α5 (green) was found. Laminin β2 (green) was observed in a basement membrane-like structure at the basal site of the retinal organoid. Laminin γ3 (green) is expressed throughout the retinal organoid with a prominent labelling at the basal site. Nuclei are counterstained with Hoechst (blue). Hoe Hoechst, Lam laminin, PanLam Pan-Laminin, Recov Recoverin. Scale bars, 20 μm

**Fig. 4:**
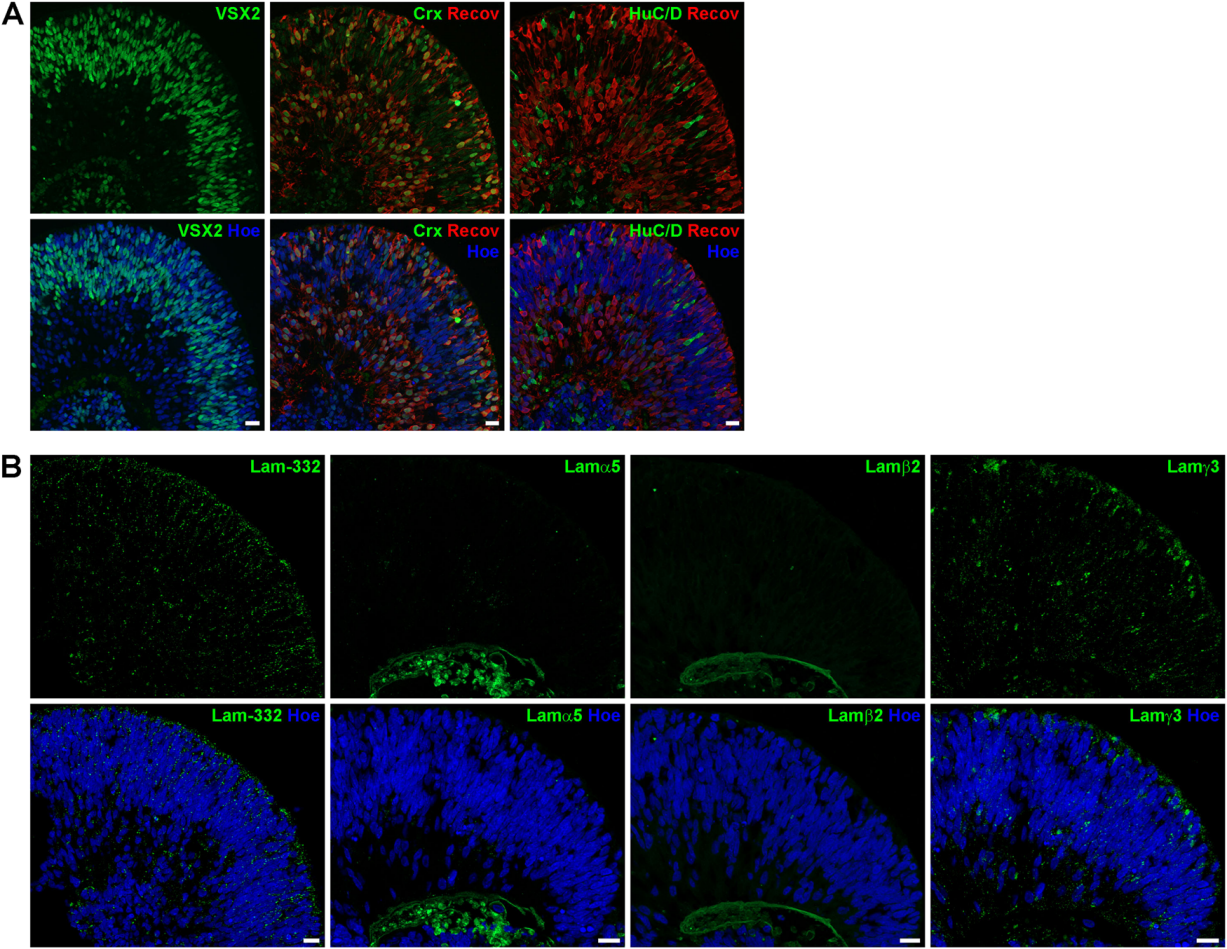
Expression of retinal markers and laminin-332, laminin α5, laminin β2 and laminin γ3 in retinal organoids derived from hESCs at day 90 of differentiation. **a)** Expression of retinal progenitor cells (VSX2, green), photoreceptors (CRX, green; Recoverin, red) and ganglion cells (HuC/D, green). **b)** Laminin-332 (green) and laminin γ3 are expressed throughout the retinal organoid with a more prominent labelling at the apical site. Laminin α5 (green) and laminin β2 (green) was observed in a basement membrane-like structure at the basal site of the retinal organoid. Nuclei are counterstained with Hoechst (blue). Hoe Hoechst, Lam laminin, Recov Recoverin. Scale bars, 20 μm

**Fig. 5:**
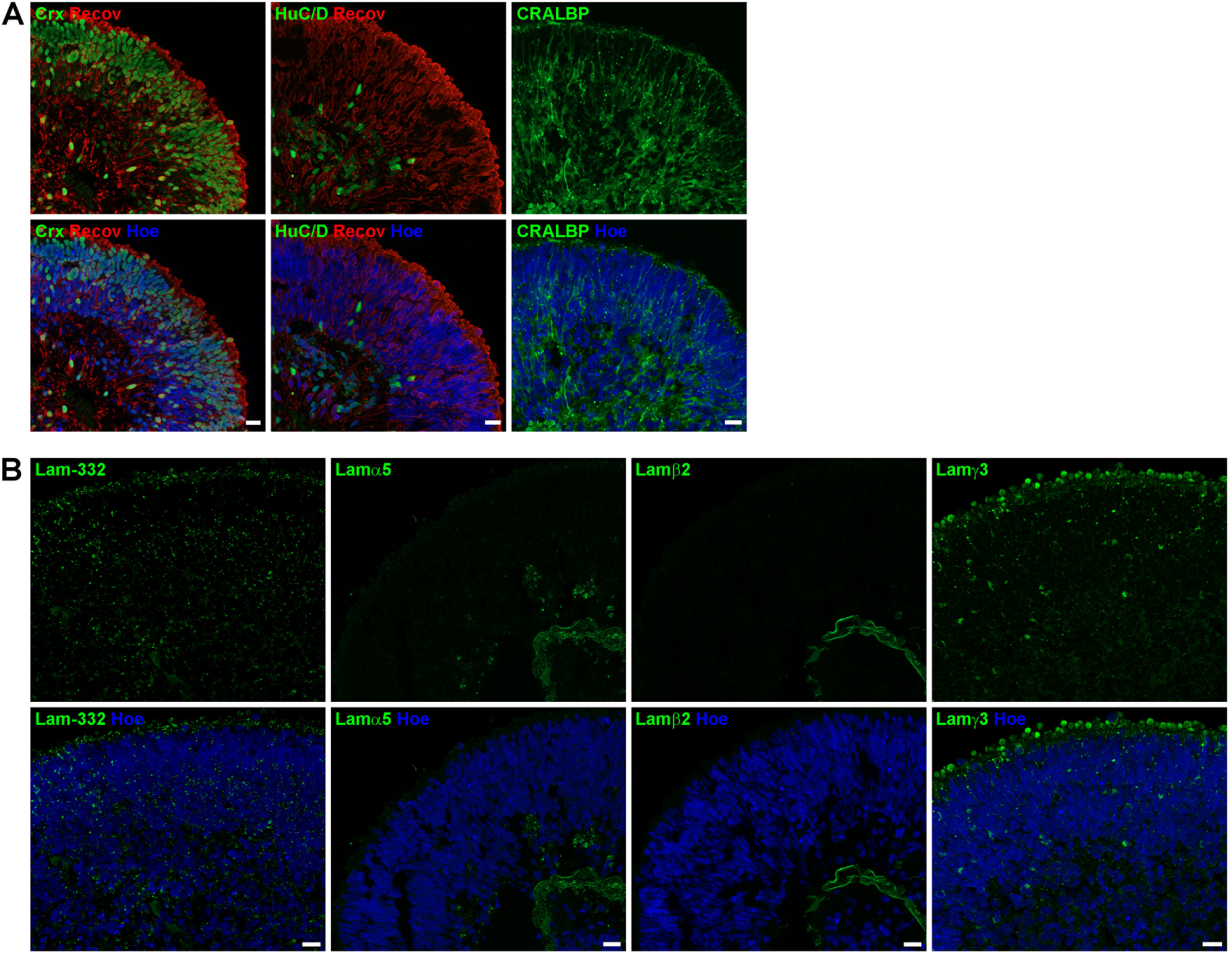
Expression of retinal markers and laminin-332, laminin α5, laminin β2 and laminin γ3 in retinal organoids derived from hESCs at day 150 of differentiation. **a)** Expression of photoreceptors (CRX, green; Recoverin, red), ganglion cells (HuC/D, green) and Müller cells (CRALBP, green). **b)** Laminin-332 (green) is expressed throughout the retinal organoids. Laminin α5 (green) and laminin β2 (green) were observed in a basement membrane-like structure at the basal site of the retinal organoid. Laminin γ3 (green) was found at the apical site of retinal organoid with a prominent labelling above cell nuclei, most likely in the IS of photoreceptors. Nuclei are counterstained with Hoechst (blue). Hoe Hoechst, IS inner segments, Lam laminin, Recov Recoverin. Scale bars, 20 μm

**Fig. 6:**
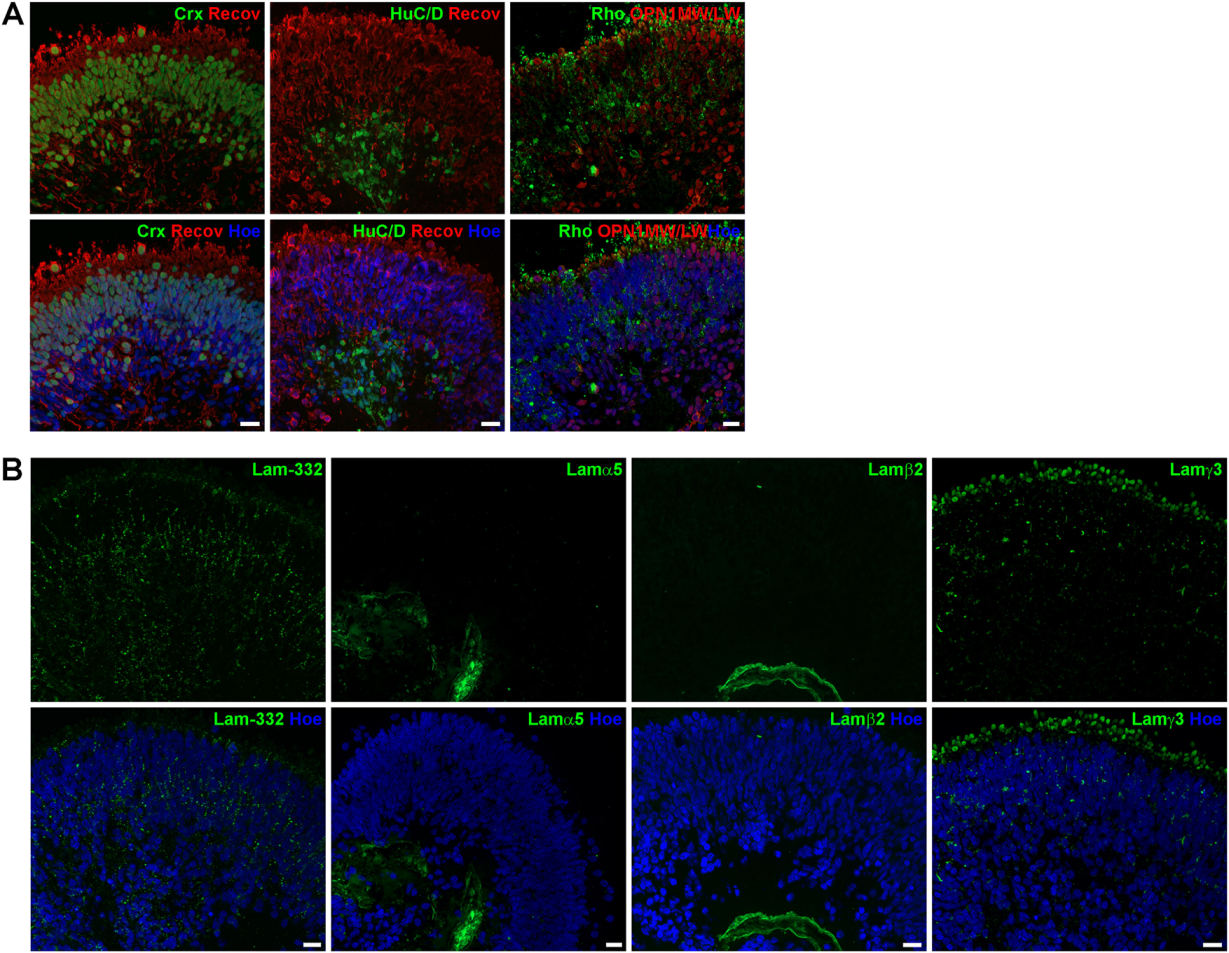
Expression of retinal markers and laminin-332, laminin α5, laminin β2 and laminin γ3 in retinal organoids derived from hESCs at day 200 of differentiation. **a)** Expression of photoreceptors (CRX, green; Recoverin, red), ganglion cells (HuC/D, green), rods (Rho, Rhodopsin, green) and cones (OPN1MW/OPN1LW, red). **b)** Laminin-332 (green) is expressed throughout the retinal organoid with a prominent labelling in the middle of the organoid. Laminin α5 (green) and laminin β2 (green) were observed in a basement membrane-like structure at the basal site of the retinal organoid. Laminin γ3 (green) was found predominantly at the apical site of retinal organoid with a prominent labelling above cell nuclei, most likely in the IS of photoreceptors. Nuclei are counterstained with Hoechst (blue). Hoe Hoechst, IS inner segments, Lam laminin, Recov Recoverin, Rho Rhodopsin. Scale bars, 20 μm

Retinal organoids were generated from hESCs/hiPSCs and collected at four different time points during differentiation to assess retinal development and laminin distribution. Retinal organoids derived from hPSCs revealed a clear apical–basal polarity as demonstrated by the apical expression of ZO-1 and basal expression of Pan-Laminin (Fig. 3a). VSX2, a marker for retinal progenitor cells, was mainly expressed in the apical layer of organoids, forming a large layer of retinal progenitor cells at days 35 and 90 of differentiation (Figs. 3a, 4a). In contrast, RGCs and amacrine cells (ACs) detected by the marker protein HuC/D were found in the basal region close to the centre of the retinal organoids at day 35 of differentiation (Fig. 3a). Immunoreactivity for CRX, a post-mitotic photoreceptor marker, was observed in cells located both in basal and apical region of retinal organoids (Fig. 3a). Recoverin (a marker for photoreceptors) was found in a few cells in the basal region of the organoids at day 35 (Fig. 3a). The expression pattern of both markers changed dramatically over time, showing an enormous increase in CRX- and Recoverin-positive cells at day 90 (Fig. 4a). At this differentiation stage, two bands of CRX- and Recoverin-positive cells were found in organoids, both in the apical and basal side (Fig. 4a). At days 150 and 200 of differentiation, cells expressing CRX and Recoverin were mainly located in the apical region of retinal organoids, forming a thick putative ONL (Figs. 5a, 6a). HuC/D-positive cells were located at the basal aspect of retinal organoids at day 150 (Fig. 5a) and 200 (Fig. 6a). At both time points, retinal organoids contained all retinal cell types including rod and cone photoreceptors (Fig. 6a), BCs, horizontal cells (HCs) and ACs (data not shown), as well as the MCs (Fig. 5a).

Laminin-332 was expressed in all developmental stages in hESC- (Figs. 3b, 6b) and hiPSC-derived retinae (Figure S6), showing a punctate pattern across the entire retinal organoids. This pattern was more intense apically at day 35 of differentiation (Fig. 3b). At day 90, laminin-332 puncta were also seen around photoreceptors as indicated by double staining with CRX (Figure S5A) in the developing ONL (Fig. 4b). Laminin-332 expression was observed throughout the neural retina on day 150 of differentiation (Fig. 5b). Apical laminin-332 immunoreactivity colocalised with RBP3, a marker specific for the IPM, confirming its expression in the IPM of retinal organoids (Figure S5B). Similar to day 150, at day 200, laminin-332 expression was observed through the neural retina, with more prominent expression in the putative developing OPL (Fig. 6b), the future site where synaptic connects between photoreceptors and second-order neurons will form.

Lamα1 was found in a basal membrane-like structure throughout differentiation in retinal organoids derived from hESCs (Figure S4). Unlike hESCs, Lamα1 expression in hiPSC-derived retinal organoids revealed a strong expression in a small number of cells in the developing ONL (Figure S6) and in a basement membrane-like structure, which started to form at days 90 and 120 of differentiation (arrowheads, Figure S6). Throughout all stages, no Lamα4 immunoreactivity was seen either in hESC- (Figure S4) or in hiPSC-derived retinae (Figure S6). Lamα5 and β2 expression shared a similar expression pattern in a basement membrane-like structure of retinal organoids derived from hESCs (Figure S3 and S6B) and hiPSCs (Figure S6), with clear Lamβ2 expression observed from day 35 and Lamα5 from day 90 onwards. Lamβ1 immunoreactivity revealed a punctate pattern apically in the developing ONL at differentiation day 90 in both hESC- and hiPSC-derived retinae (Figure S4B, S6B). Later at day 200 for hESC-derived retinal organoids and at day 120 for hiPSC-derived retinae, Lamβ1 expression was observed in a basal basement membrane-like pattern at the centre of organoids (Figure S4D, S6C). Lamβ2 and γ1 were found in a basal basement membrane-like structure in organoids derived from both hPSC cell lines throughout development (Figs. 3, 6b, S4,S6). Double staining with Lamβ2 and Collagen IV, a component of basement membranes, revealed colocalisation, confirming that Lamβ2 is expressed in a basement membrane of organoids (Figure S5A).

At all developmental stages, Lamγ3 showed a punctate pattern across the entire retinal organoid derived from hESCs (Figs. 3, 6b) and hiPSCs (Figure S6). At day 35 of differentiation, this pattern was more prominent in the basal aspect of the neural retina (Fig. 3b), whereas at day 90 Lamγ3 immunoreactivity showed weak basal expression (Fig. 4b) and instead revealed strong expression at the apical surface where developing photoreceptors are located (Figure S5A). Lamγ3 expression in hiPSC-derived retinal organoids showed a comparable distribution pattern to retinal organoids derived from hESCs (Figure S6). At day 150, Lamγ3 was found in the layer apical to the developing ONL in hESCs-derived retinal organoids (Fig. 5b), indicating expression in the developing inner segments (ISs) of photoreceptors. In addition, double-labelling with antibodies against Lamγ3 and RBP3 confirmed that Lamγ3 is also expressed in the IPM (Figure S5B). At day 200, the Lamγ3 immunoreactivity revealed the same pattern as in day 150, although it was stronger than at earlier differentiation time points (Fig. 6b).

### Effects of neutralising Laminin γ3 in human retinal development

**Fig. 7:**
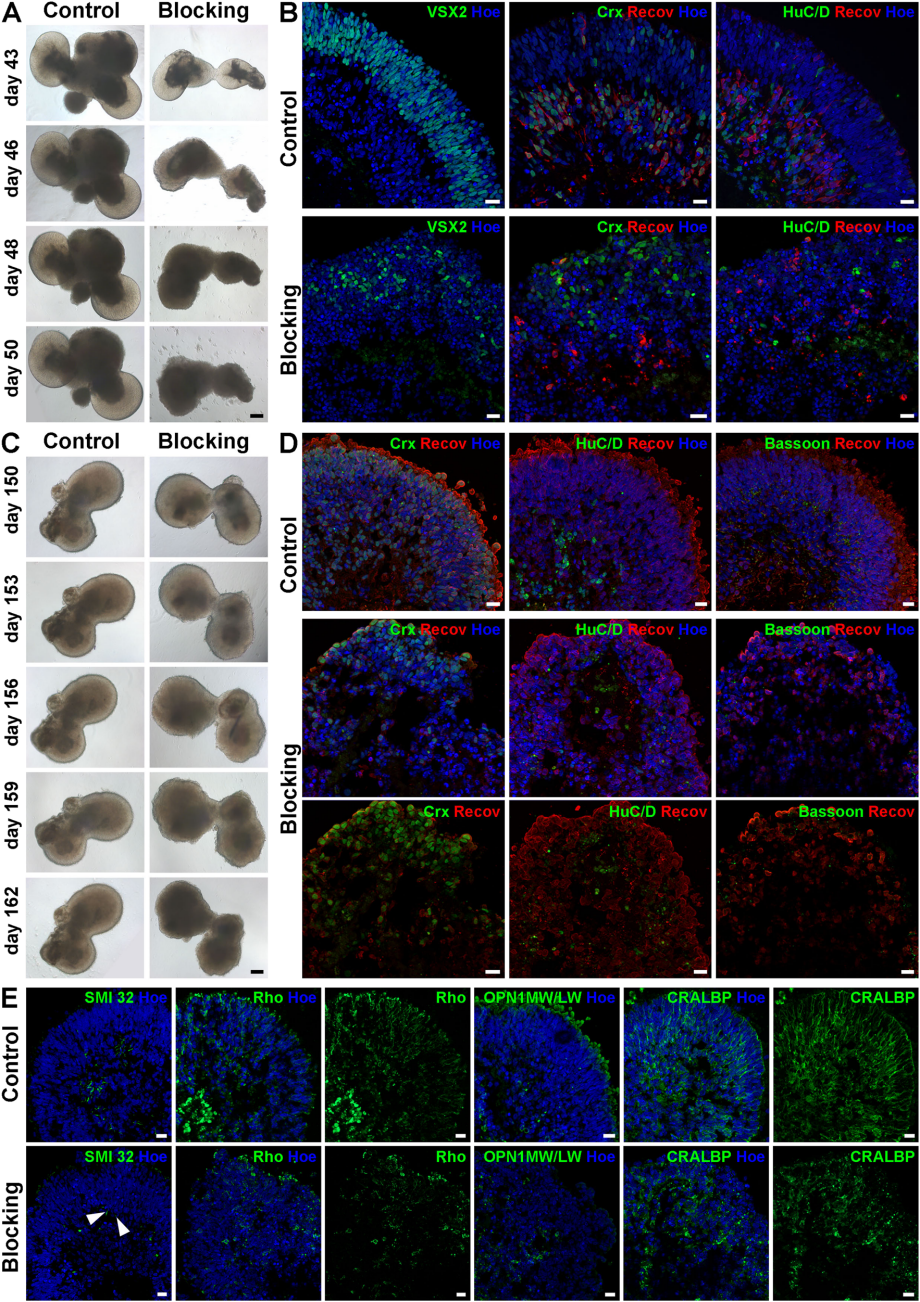
Blocking of laminin γ3 in retinal organoids derived from hESCs at day 43 and day 150 of differentiation. **a)** Brightfield images revealed a degeneration of bright phase retinal neuroepithelial structures at the apical edge of the organoids in the blocking condition over time in the early blocking experiment (day 43). **b)** Expression of retinal progenitor cells (VSX2, green), photoreceptors (CRX, green; Recoverin, red) and ganglion cells (HuC/D, green) showed a reduction in cell number and a disruption of the lamination in retinal organoids after blocking of laminin γ3 at day 43 of differentiation. **c)** Brightfield images indicated a degeneration of the retinal neuroepithelial structures at the edge of organoids over time upon blocking of laminin γ3 at day 150 of differentiation. **d**) Expression of photoreceptors (CRX, green; Recoverin, red) indicated the lamination loss in retinal organoids after blocking of laminin γ3 at day 150 of differentiation. Expression of ganglion cells (HuC/D; green) were absent in the blocking condition. Bassoon (green) expression is significantly decreased in blocked-retinal organoids. **e)** After laminin γ3 blocking only a few ganglion cell dendrites detected by SMI-32 (green) were still present (arrowheads). Expression of rods (Rho, green), cones (OPN1MW/OPN1LW, green) and Müller cells (CRALBP, green) revealed the disrupted lamination of retinal organoids and a reduction in their cell number at day 150 of differentiation. Nuclei are counterstained with Hoechst (blue). Hoe Hoechst, Recov Recoverin, Rho Rhodopsin. Scale bars, 50 μm for **a** and **c**; 20 μm for **b**, **d**, **e**

To investigate the role of Lamγ3 during hESC differentiation, an antibody was used to neutralise Lamγ3 function at two different developmental time points. Blocking of Lamγ3 at day 43 induced degeneration of the bright phase retinal neuroepithelial structures observed at the periphery of organoids over time compared with control retinal organoids (Fig. 7a). The control organoids showed the expected expression of Recoverin, CRX and VSX2, including a thick apical layer of VSX2-positive cells and almost all Recoverin- and CRX-positive cells in the inner retina (Fig. 7b, control). Although blocking Lamγ3 still resulted in detection of the aforementioned markers, the retina-like structural organisation was disrupted, showing disorganised and reduced expression of VSX2, Recoverin and CRX, as well as HuC/D (Fig. 7b) and SMI-32 (Figure S7B). These findings were confirmed by qRT-PCR results, which revealed a decrease in *VSX2* expression and a significant reduced expression of *CRX* and *MATH5* (an RGC marker) under blocking conditions (Figure S7A). Expression of caspase-3, an apoptosis marker, was increased under blocking condition (31.34 ± 3.51%) compared with control group (8.11 ± 1.38%; Figure S7B).

Similar findings were obtained by blocking Lamγ3 later at day 150. Under blocking conditions, the retinal neuroepithelium at the periphery of organoids degenerated over time while that of control organoids remained intact (Fig. 7c). Ganglion cell bodies (detected by HuC/D immunostaining) and ganglion cell dendrites (detected by SMI-32 immunostaining) were observed in the inner retina in control organoids (Figs. 7d, e). No ganglion cell bodies were detected (Fig. 7d) under the blocking conditions, but a few putative remaining dendrites were still found (arrowheads, Fig. 7e). In accordance, qRT-PCR data revealed a significant reduction in the expression of *MATH5* (Figure S7C). Caspase-3 expression was also increased under blocking conditions (20.99 ± 5.4%) compared with control group (1.1% ± 0.16). The Caspase-3 positive cells were found throughout organoids, including the basal layer where ganglion cells are located (Figure S7D). Together these findings indicate that that ganglion cells may undergo apoptosis in response to Lamγ3 neutralisation. The number of photoreceptor precursors decreased in Lamγ3 blocked cultures as demonstrated by an immunolabelling with Recoverin and CRX (Fig. 7d). Moreover, in the blocking condition photoreceptors were disorganised compared with those in control organoids where developing photoreceptors formed a thick putative ONL (Fig. 7d). This was also reflected in the expression pattern of Rhodopsin, which was reduced and disorganised in organoids incubated with blocking antibody to Lamγ3 (Fig. 7e). Opsin immunoreactivity was weak and only found in the basal part of the retinal organoids for OPN1MW/OPN1LW (Fig. 7e) and almost absent for OPN1SW under blocking conditions (Figure S7D). In contrast, control organoids revealed a prominent OPN1MW/OPN1LW immunolabelling in putative photoreceptor IS adjacent to the ONL (Fig. 7e) and OPN1SW expression in cells located in the basal part of retinal organoids (Figure S7D). These findings were confirmed at gene expression level, showing a significant decrease in the expression of Recoverin, Rhodopsin and Opsins (Figure S7C). In control, retinal organoids punctate Bassoon labelling was found in the middle and in the basal part of organoids colocalised with Recoverin, suggesting that structural elements of the ribbon synapses are present (Fig. 7d). This pattern was significantly reduced under blocking conditions (Fig. 7d). In addition, Syntaxin immunoreactivity was also reduced under blocking conditions (arrowhead, Figure S7D), which was also corroborated by qRT-PCR data (Figure S7C). CRALBP (MCs/RPE), AP2α (ACs) and Prox1 (HCs) were used to assess any effects of blocking Lamγ3 on other retinal cell types. In control retinal organoids, CRALBP immunoreactivity spanned the retinal neuroepithelium in a radial pattern (Fig. 7e), whereas this pattern was disrupted in retinal organoids under blocking conditions (Fig. 7e). These changes were also reflected at gene expression level (Figure S7C). The number of AP2α-positive cells was reduced in retinal organoids in the blocking condition compared with control organoids (Figure S7D). In accordance, a significant downregulation of *AP2α* was observed by qRT-PCR (Figure S7C). No changes between control and blocking condition were found for HCs at protein (Figure S7D) and gene expression level (data not shown). Interestingly, RBP3, a marker for the IPM, showed a significant reduction at both protein and gene expression level (Figure S7C, D).

## Discussion

This study provides a detailed and comprehensive expression analysis for the majority of existing laminin chains in adult mouse, macaque and human retina, developing human eye and retinal organoids derived from hPSCs. All laminin chains displayed unique and distinct temporal and spatial distribution in the adult retina and developing human eye, revealing expression of Lamα1, α5, β1, β2 and γ1 predominantly in retinal basement membranes and expression of laminin-332 and Lamγ3 throughout the retina. hPSC-derived retinal organoids mimicked laminin expression during early human retinal development. Furthermore, the study demonstrates for the first time that Lamγ3 is essential for retinal lamination, photoreceptor organisation, differentiation of RGCs, as well as synapse stability during human retinogenesis.

Lamγ3, is broadly expressed in skin, heart, lung, reproductive tracts, brain and retina^9,10^. Although a single ablation of either Lam γ3 or Lamβ2 causes minimal or almost no changes in retinal morphology in other studies^24,48,49^, double-knockout of both laminin chains results in defects of the ILM formation during retinal development, leading to retinal dysplasia^24^. Furthermore, a deletion of both Lamβ2 and γ3 was shown to impair MC maturation and physiological function^50^. We used the retinal organoid model to block the function of Lamγ3 both early (day 43) and late (day 150) time points in development when retinal lamination is more advanced and multiple retinal cell types are present. Blocking of Lamγ3 function early in development resulted in disruption of laminar organisation and a significant reduction in the expression of retinal progenitor marker (VSX2), photoreceptor marker (CRX and Recoverin) and ganglion/ACs (HuC/D). Later in development similar results were obtained, showing disrupted laminar organisation and reduced expression of specific marker proteins for photoreceptors, ACs and the synaptic markers. Amacrine and ganglion cell marker protein HuC/D was absent and only a few dendritic structures were observed in blocked-retinal organoids, suggesting an essential role for this laminin chain in ganglion cell differentiation. The ribbon synapse marker protein, Bassoon, and Syntaxin, a presynaptic marker protein, were significantly reduced upon Lamγ3 blocking, suggesting that Lamγ3 might be involved in synapse stability/formation at this stage of development. The disorganisation of MCs, which form the ILM by their end-feet processes, could be a reason for the reduction in RGCs. Supporting evidence come from Halfter et al. ^51^, showing that defects in, or absence of an ILM, leads to apoptosis of RGCs.

Ribbon synapse formation during retinal development was also affected after blocking of Lamγ3. To date only Lamβ2 has been associated with the formation of synapses either in the peripheral nervous system ^52,53^ or central nervous system, including the retina^31,54,55^. Mouse lacking Lamβ2 showed dysmorphic photoreceptor synapses^56^. Recently, it was shown that interaction between Lamβ2 and integrins such as integrin α3β1, is important for synaptic ribbon anchoring at the photoreceptor synapse and that the dystrophin/dystroglycan complex might play a key role in synaptic formation^55^. Integrins act as receptors for many laminins^57^. As Lamγ3 lacks the binding domain for integrins^58^ it is impossible to act via integrins; however, Lamγ3 may interact via non-integrin binding proteins such as entactins and dystroglycans, which have been shown to be important for the photoreceptor synapses^59^. Our data suggest that in addition to Lamβ2, Lamγ3 plays a key role in the formation and/or stabilisation of the photoreceptor synapses, acting perhaps via a non-integrin binding protein such as dystroglycan.

In summary, our data provide a comprehensive laminin expression analysis in mouse, macaque and developing and adult human retina and indicate a novel function for Lamγ3 in retinal lamination, organisation of the ONL and differentiation of RGCs in hPSC-derived retinal organoids.

## Electronic supplementary material

Supplement Figure Legends

Figure S1

Figure S2

Figure S3

Figure S4

Figure S5

Figure S6

Figure S7

List of primary antibodies used for immunohistochemistry

List of primers used for qRT-PCR

Laminin expression in adult mouse, macaque and human retina

Laminin expression in human developing eye
