# Supplementary material for: Laminin γ3 plays an important role in retinal lamination, photoreceptor organisation and ganglion cell differentiation
Source: Cell Death Dis. 2018 May 23;9(6):615. doi: 10.1038/s41419-018-0648-0 (PMC5966411; doi:10.1038/s41419-018-0648-0)
Supplement: Supplementary file 1 — Supplement Figure Legends [file 41419_2018_648_MOESM1_ESM.docx]

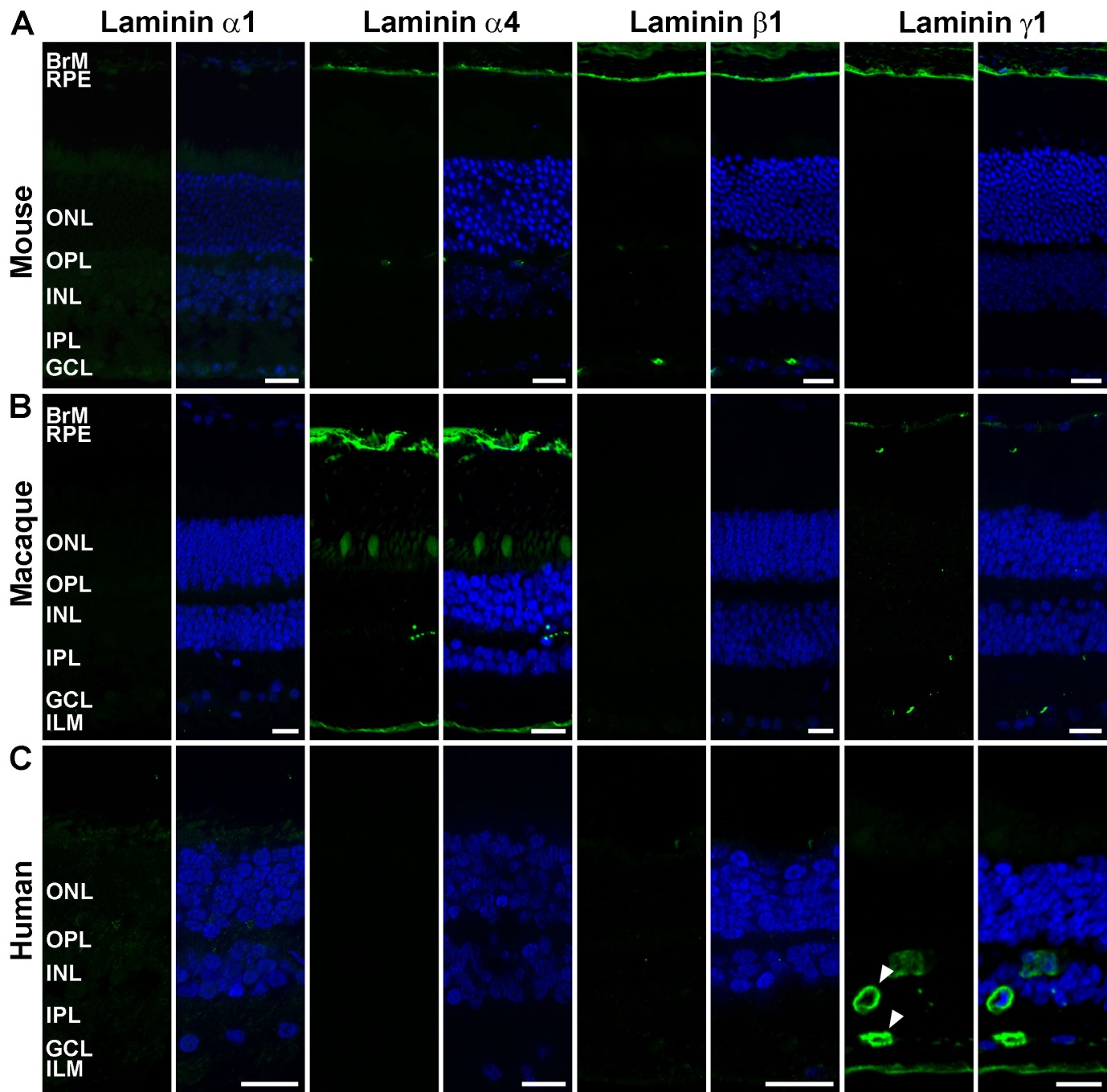


**Figure S1.** Expression of laminin α1, laminin α4, laminin β1 and laminin γ1 in adult mouse (A), macaque (B) and human retina (C). No expression of laminin α1 (green) in the mouse retina (A), in the macaque retina (B) and in the human retina (C). Laminin α4 (green) is expressed in BrM of mouse retina (A) and in BrM and ILM of the macaque retina (B) and not in the human retina (C). Laminin β1 (green) was found in BrM of mouse retina (A), and not in of macaque (B) and human retina (C). Nuclei are counterstained with Hoechst (blue). Arrowheads indicate expression of laminin γ1 in blood vessels in the human retina (C). Nuclei are counterstained with Hoechst (blue). Abbreviations: BrM, Bruch’s membrane; RPE, retinal pigment epithelium; ONL, outer nuclear layer; OPL, outer plexiform layer; INL, inner nuclear layer; IPL, inner plexiform layer; GCL, ganglion cell layer; ILM, inner limiting membrane; Hoe, Hoechst. Scale bars, 20 μm.


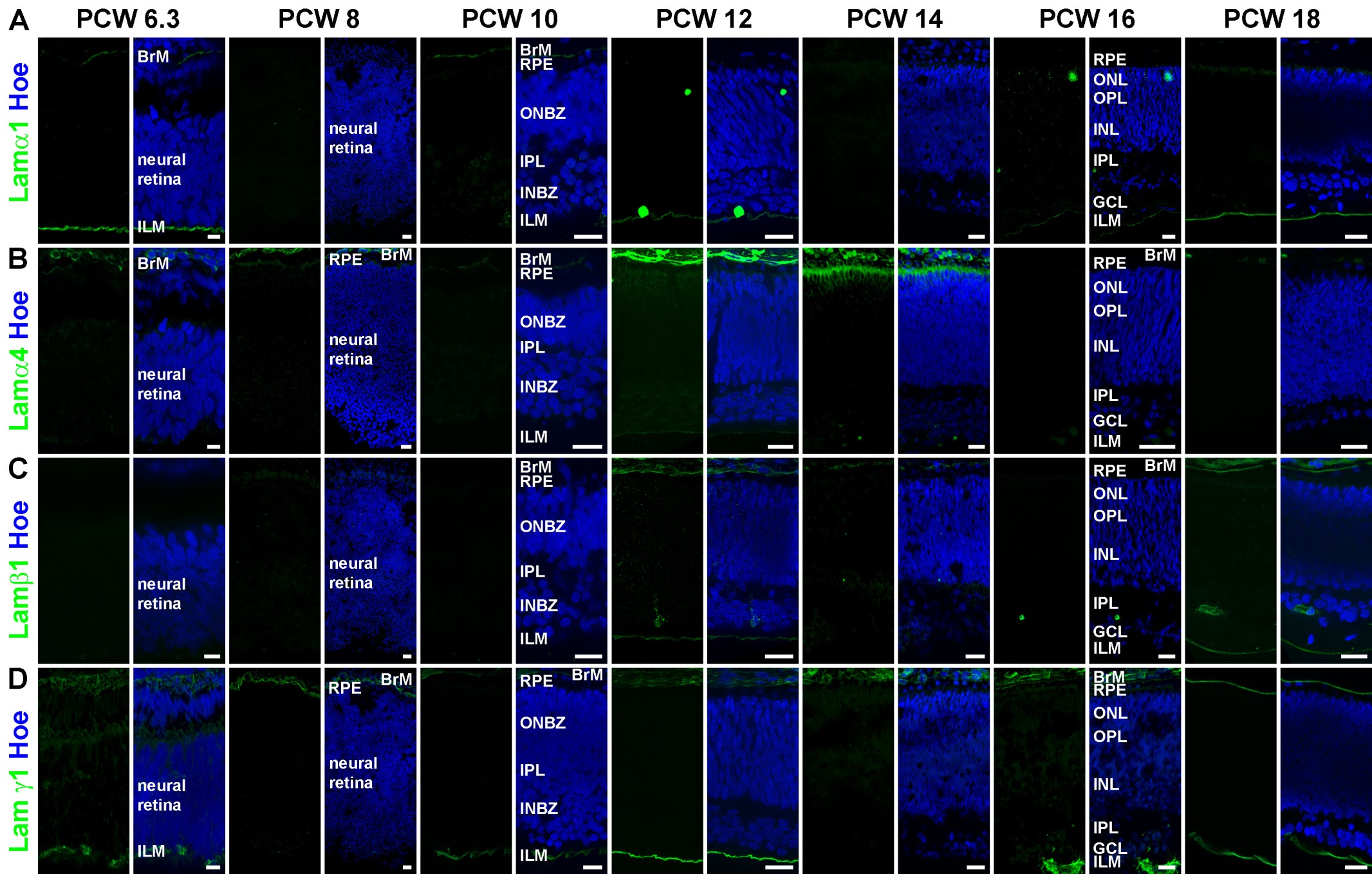


**Figure S2.** Expression of laminin α1 (A), laminin α4 (B), laminin β1 (C) and laminin γ1 (D) in the developing human retina. A: Laminin α1 (green) is expressed in BrM at 6.3 and 10 PCW and in ILM at 6.3, 12, 16 and 18 PCW. B: Laminin α4 (green) was found in BrM, IPM and ILM at 12 PCW and in IPM and ONBZ at 14 PCW. C: Laminin β1 (green) was observed in BrM from 12 PCW onwards, except 16 PCW and in the ILM at 12 and 18 PCW. D: Laminin γ1 (green) is expressed in the ILM and across the retina at 6.3 PCW, in BrM from 8 PCW onwards, except for 10 PCW and in ILM from 10 PCW onwards, except for 14 PCW. Nuclei are counterstained with Hoechst (blue). Abbreviations: BrM, Bruch’s membrane; RPE, retinal pigment epithelium; ONBZ, outer neuroblastic zone; ONL, outer nuclear layer; OPL, outer plexiform layer; INBZ, inner neuroblastic zone; INL, inner nuclear layer; IPL, inner plexiform layer; GCL, ganglion cell layer; ILM, inner limiting membrane; Hoe, Hoechst; Lam, laminin; PCW, post conceptual week. Scale bars, 20 μm.


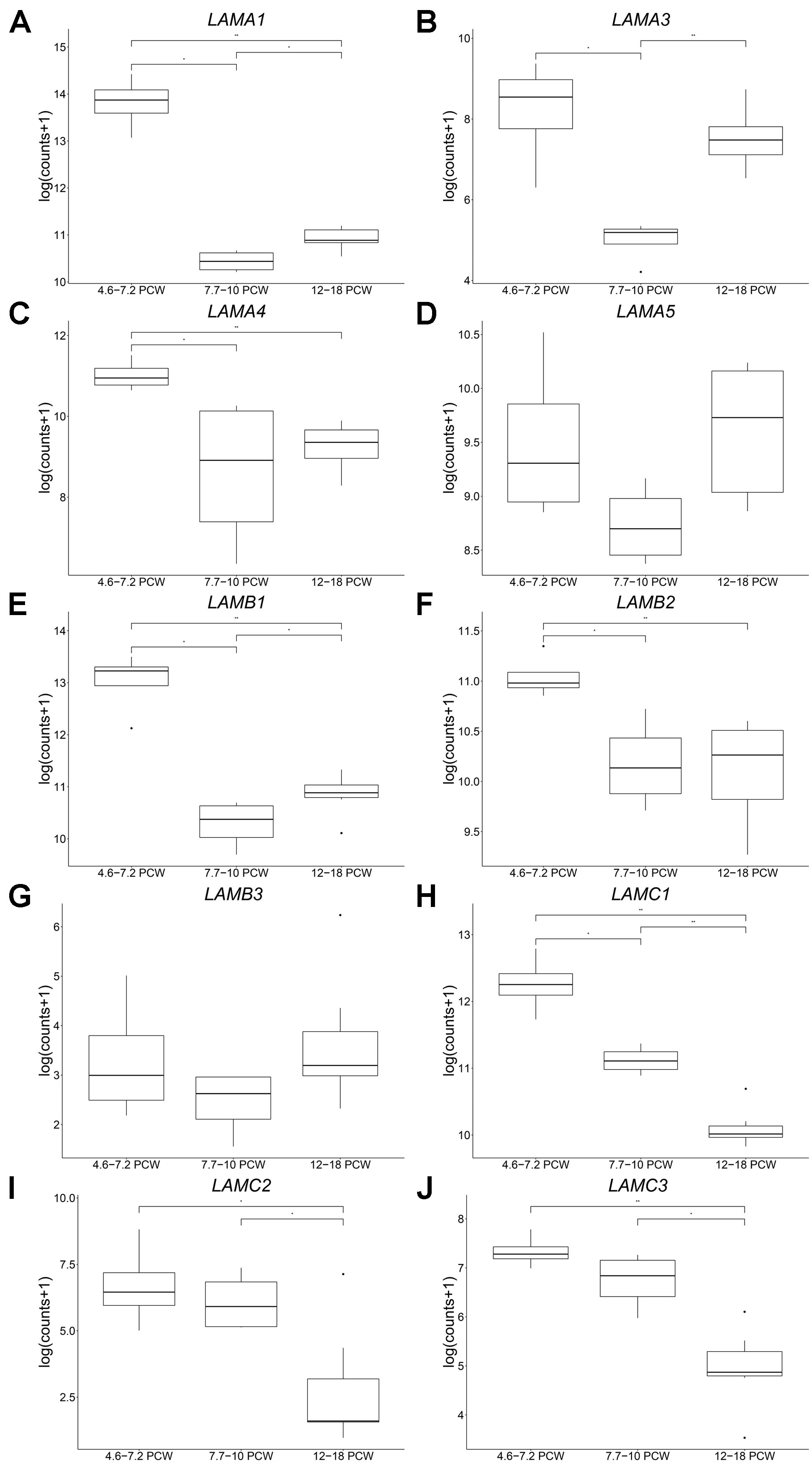


**Figure S3:** Analysis of laminin chain expression during the development of human retina.  Box-and-whisker plots were obtained by grouping the samples into three developmental windows: 4.6-7.2 PCW, 7.7-10 PCW and 12-18 PCW. Individual boxes quantify the distribution of the expression of selected genes in these associated windows. To this end, the logarithm of the counts (normalised using the DESeq2 package in the Bioconductor infrastructure) was computed. For statistical assessment of differences between the epochs, the Mann-Whitney U test was used. For statistical assessment of differences between the developmental windows, the Mann-Whitney U test (Wilcoxon Rank-Sum test) was used. Asterisk = p-value < 0.05, double asterisk = p-value < 0.01. All figures were created using Rstudio 1.1.419 and Ubuntu16.04 as operating system. Abbreviations: PCW, post conceptual week.


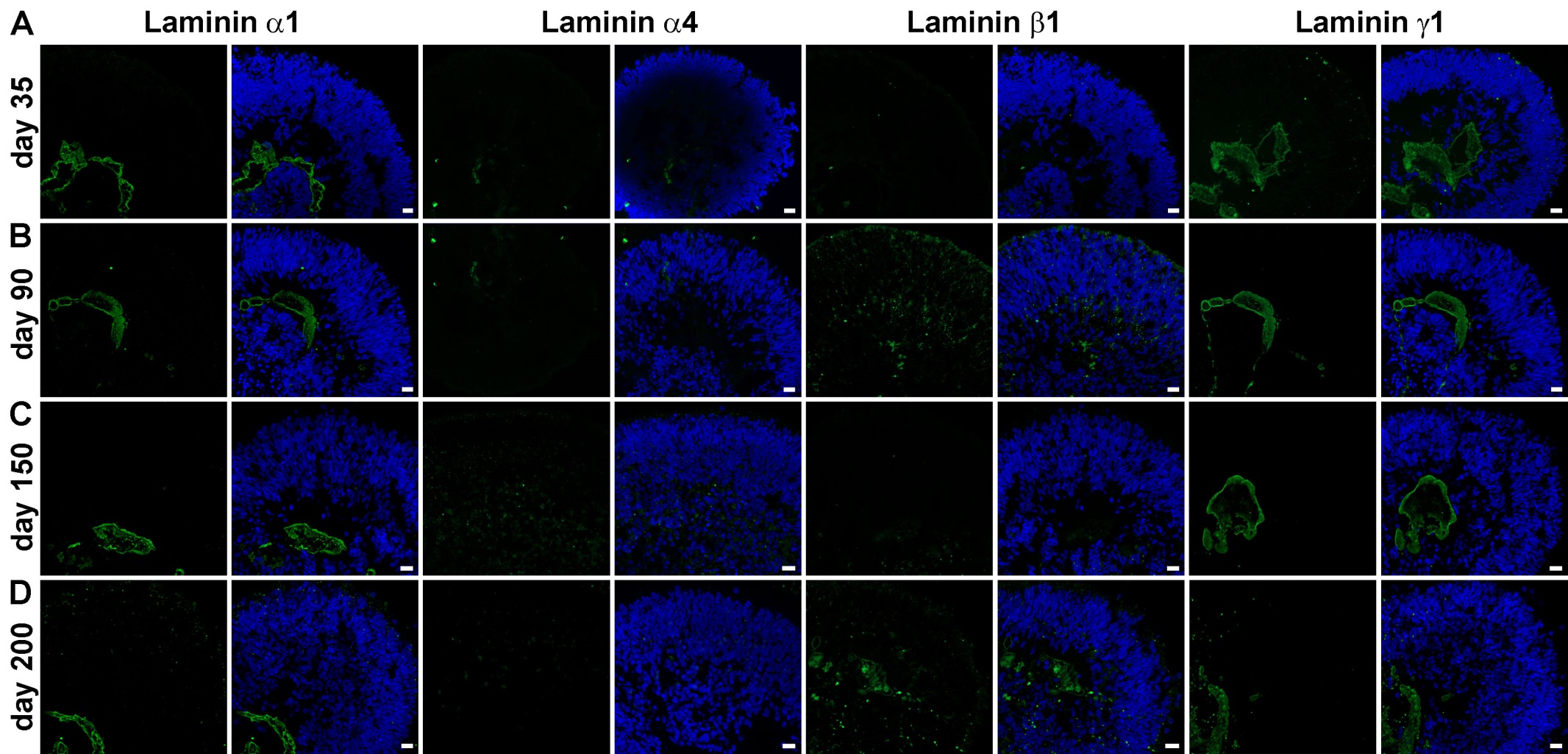


**Figure S4.** Expression of laminin α1, laminin α4, laminin β1 and laminin γ1 in retinal organoids derived from hESCs at day 35 (A), 90 (B), 150 (C) and 200 (D) of differentiation. Laminin α1 (green) is expressed in a basement membrane-like structure in the center of retinal organoids throughout all differentiation stages. No laminin α4 immunoreactivity (green) was found at all differentiation stages in the retinal organoid. A punctate laminin β1 (green) expression pattern was observed apically and basically in retinal organoids at day 90 and changed later at day 120, being expressed in a basement membrane-like structure in the center of organoids. Laminin γ1 (green) is expressed in a basement membrane-like structure in the basal site of retinal organoids throughout all stages. Nuclei are counterstained with Hoechst (blue). Scale bars, 20 μm.


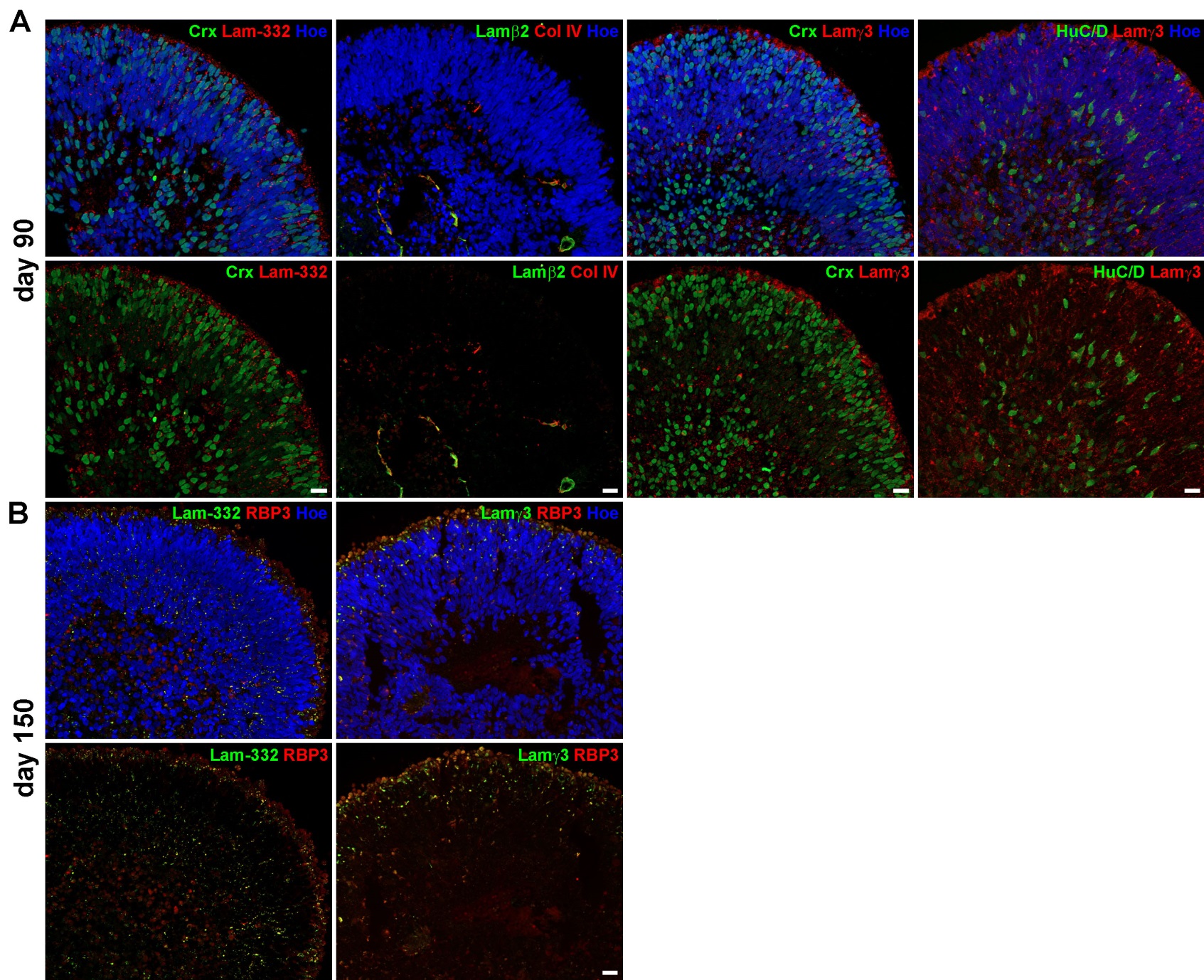


**Figure S5.** Immunohistochemistry double-labeling for laminin-332, laminin β2 and laminin γ3 in retinal organoids derived from hESCs at day 90 (A) and 150 (B) of differentiation. A: Laminin-332 (red) is expressed around photoreceptors labeled with CRX (green). Laminin β2 (green) showed co-localization with collagen-IV (red). Laminin γ3 is expressed around photoreceptors labeled with CRX (green) and ganglion cells marked with HuC/D (green). B: Laminin-332 and laminin γ3 are co-localized with RBP3, a marker for the interphotoreceptor matrix. Nuclei are counterstained with Hoechst (blue). Abbreviations: Col IV, collagen-IV; Hoe, Hoechst; Lam, laminin. Scale bars, 20 μm.


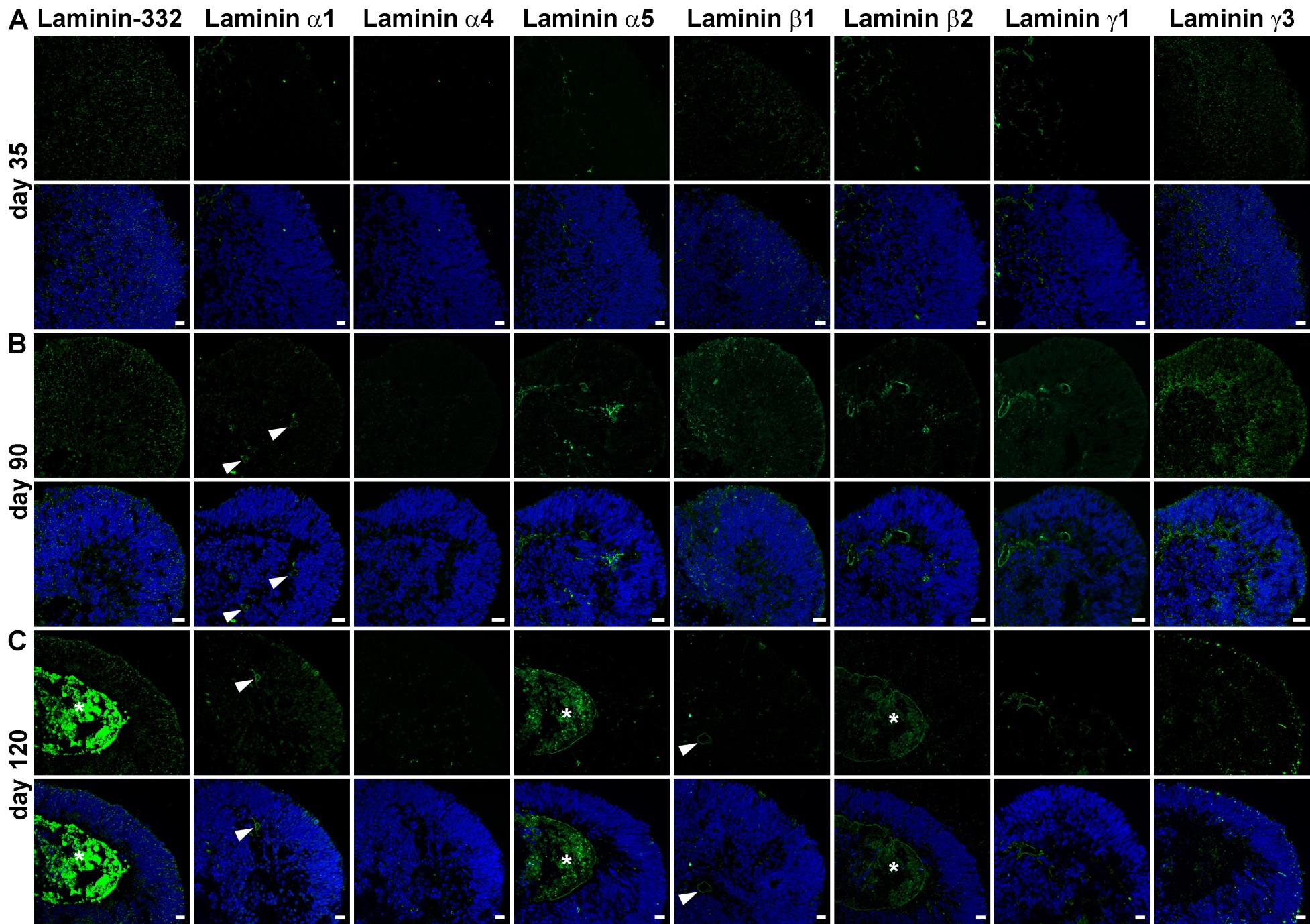


**Figure S6.** Expression of laminin-332, laminin α1, laminin α4, laminin α5, laminin β1, laminin β2, laminin γ1 and laminin γ3 in retinal organoids derived from hiPSCs at day 35 (A), 90 (B) and 120 (C) of differentiation. Laminin-332 (green) and laminin γ3 (green) are expressed throughout the retinal organoid at all differentiation stages. Laminin α1 (green) was found throughout the retina and in a basement membrane-like structure in the basal site of the organoid at day 90 and 120 of differentiation (arrowheads). No laminin α4 immunoreactivity (green) was observed at all differentiation stages in the retinal organoid. Laminin α5 (green), laminin β2 (green) and laminin γ1 (green) were expressed in a basement membrane-like structure at the basal site of organoids from day 90 of differentiation onwards. Laminin β1 (green) expression changed from being throughout the neural retina at day 90 of differentiation to expression in basement membrane-like structure at day 120 of differentiation (arrowheads). Stars indicate non-specific background staining in the center of the retinal organoids. Nuclei are counterstained with Hoechst (blue). Scale bars, 20 μm.


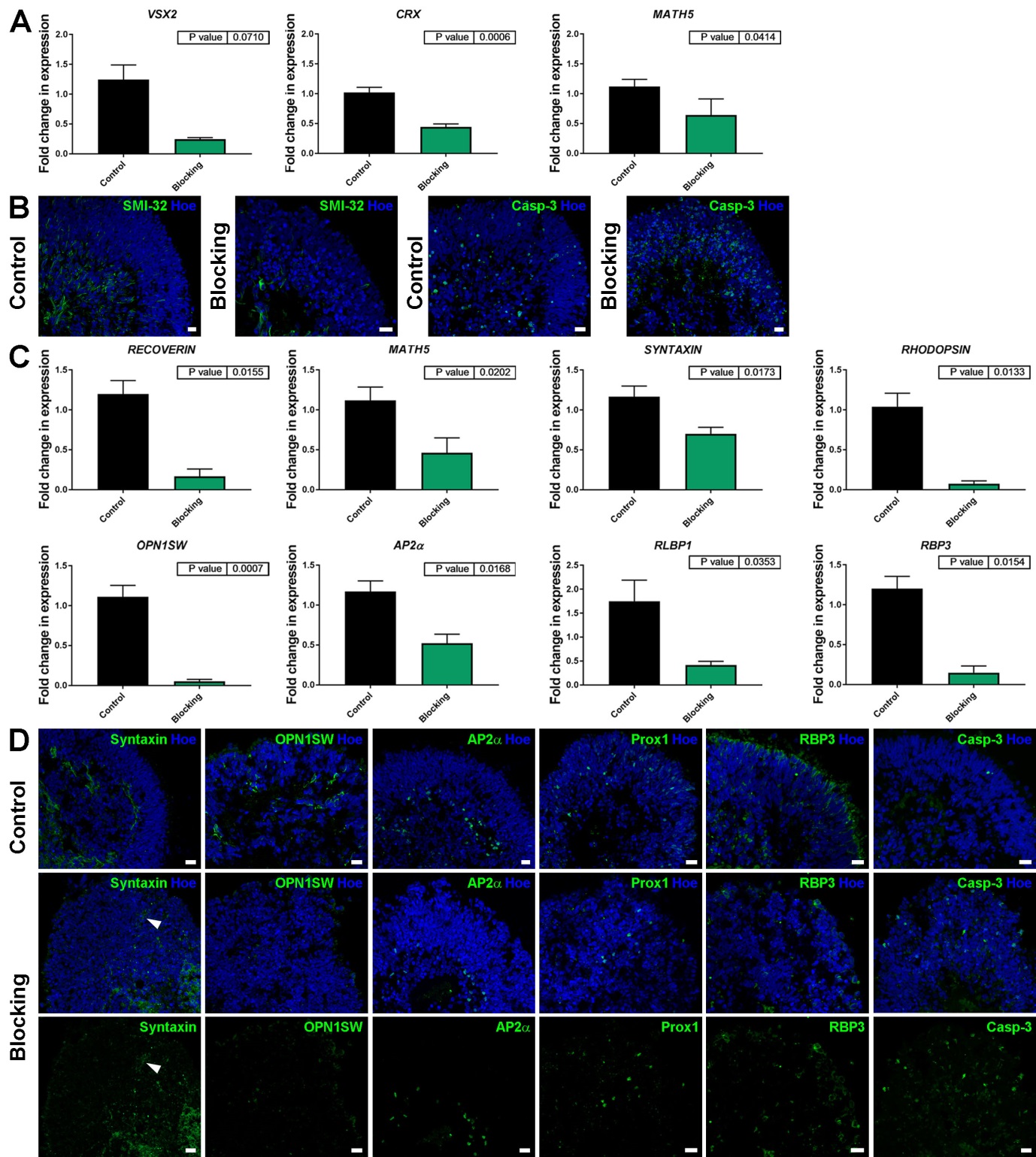


**Figure S7:** Blocking of laminin γ3 in retinal organoids derived from hESCs at day 43 (A,B) and day 150 (C,D) of differentiation. A: qRT-PCR analysis revealed a decrease of retinal progenitor precursor (*VSX2*) expression and a significant reduction of photoreceptors (*CRX*) and retinal ganglion cells (*MATH5*) upon laminin γ3 blocking at day 43 of differentiation. B: Expression of SMI-32(green) was reduced and Caspape-3 expression (Casp-3; green) was increased under blocking condition at day 43. C: qRT-PCR analysis indicated a significant reduction of Recoverin (photoreceptors), *MATH5* (ganglion cells), Syntaxin (presynaptic protein), Rhodopsin (rods), *OPN1SW* (S-Cones), *AP2α* (amacrine cells), *RLBP1* (Müller cells) and RBP3 (IPM protein) expression. D: Expression of Syntaxin (green), OPN1SW (short wavelength cones; green), amacrine cells (AP2α; green), horizontal cells (Prox1; green) and RBP3 (green) indicated a reduction in expression after laminin γ3 blocking at day 150 of differentiation. Caspase-3 expression (Casp-3; green) showed an increase under laminin γ3 blocking at day 150. Arrowhead indicates remaining Syntaxin immunoreactivity in retinal organoids. Nuclei are counterstained with Hoechst (blue). qRT-PCR results are presented as mean ± SEM. Abbreviations: Casp-3, Caspase-3; Hoe, Hoechst; IPM, interphotoreceptor matrix. Scale bars, 20 μm.
