# Supplementary figures and images for: Laminin γ3 plays an important role in retinal lamination, photoreceptor organisation and ganglion cell differentiation
Source: Cell Death Dis. 2018 May 23;9(6):615. doi: 10.1038/s41419-018-0648-0 (PMC5966411; doi:10.1038/s41419-018-0648-0)

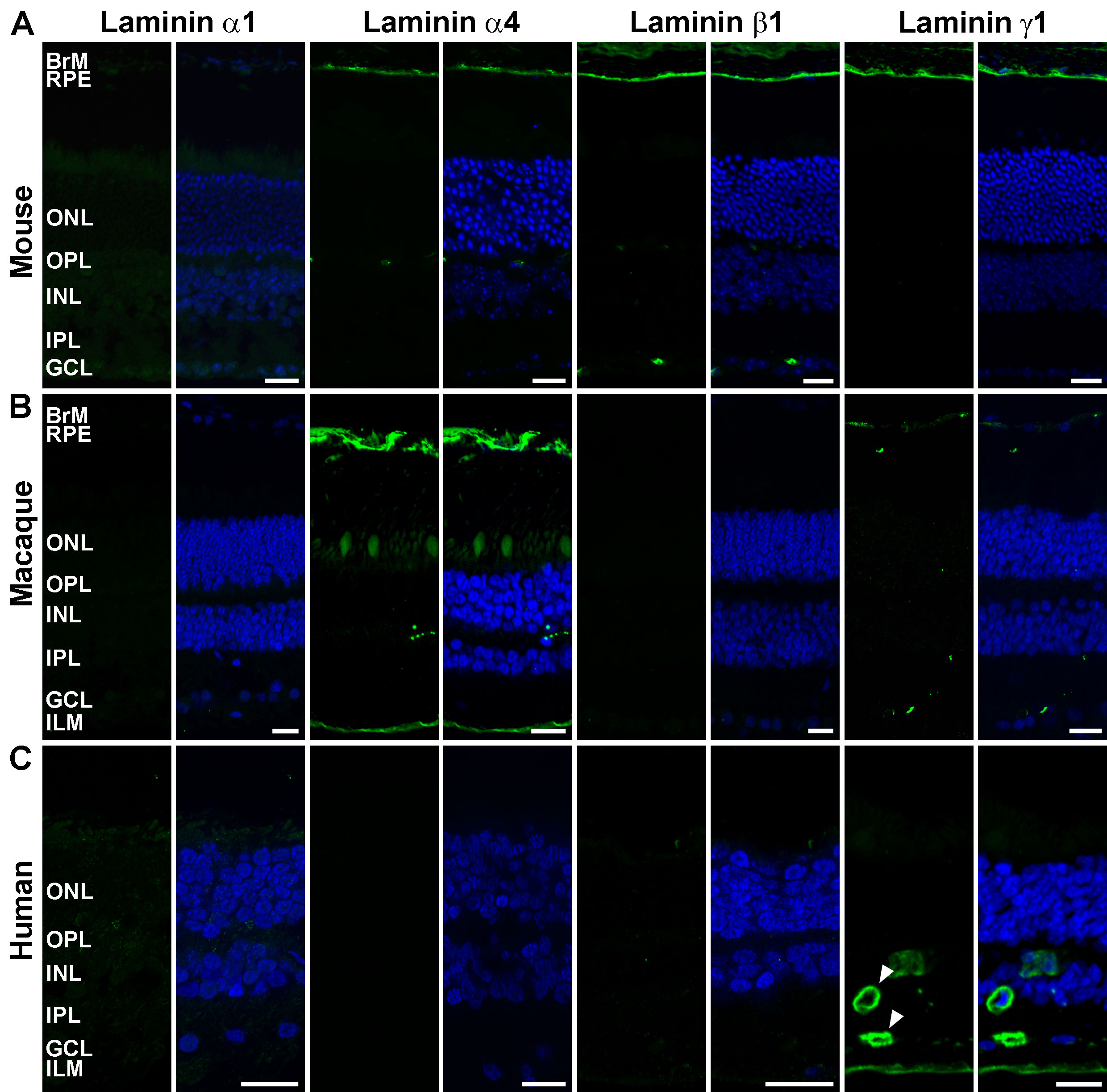

Supplement: Supplementary file 2 — Figure S1 [file 41419_2018_648_MOESM2_ESM.jpg]

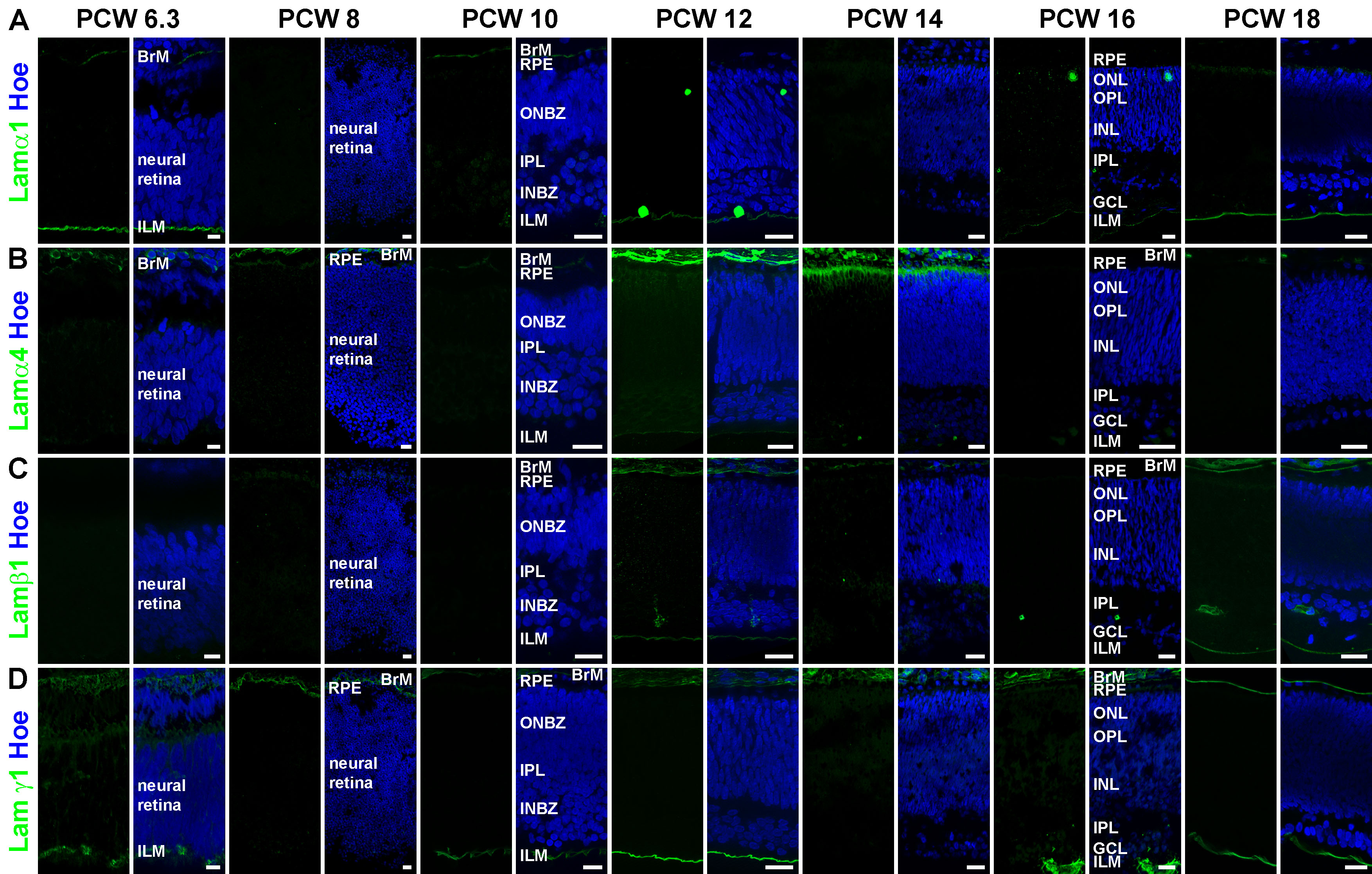

Supplement: Supplementary file 3 — Figure S2 [file 41419_2018_648_MOESM3_ESM.jpg]

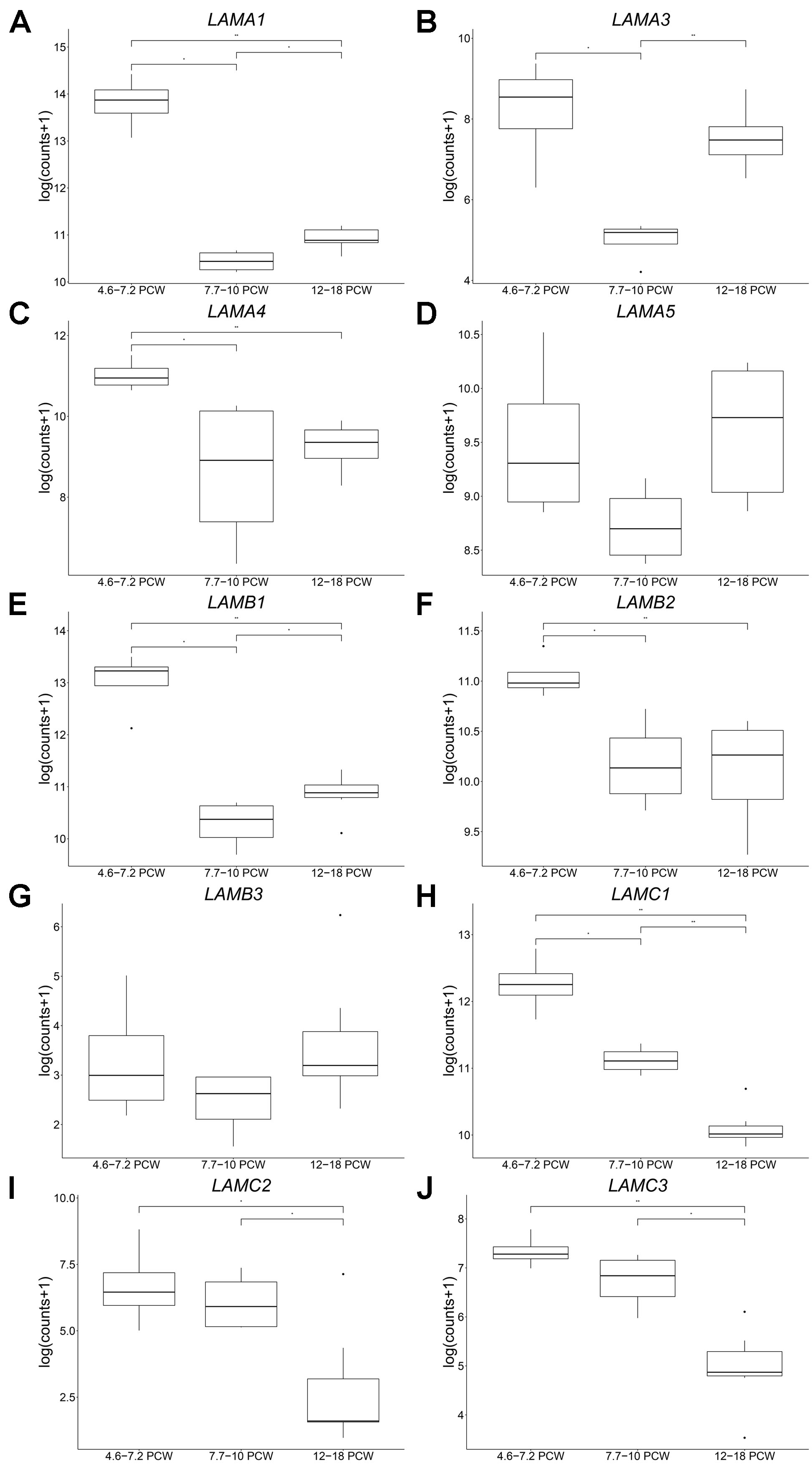

Supplement: Supplementary file 4 — Figure S3 [file 41419_2018_648_MOESM4_ESM.jpg]

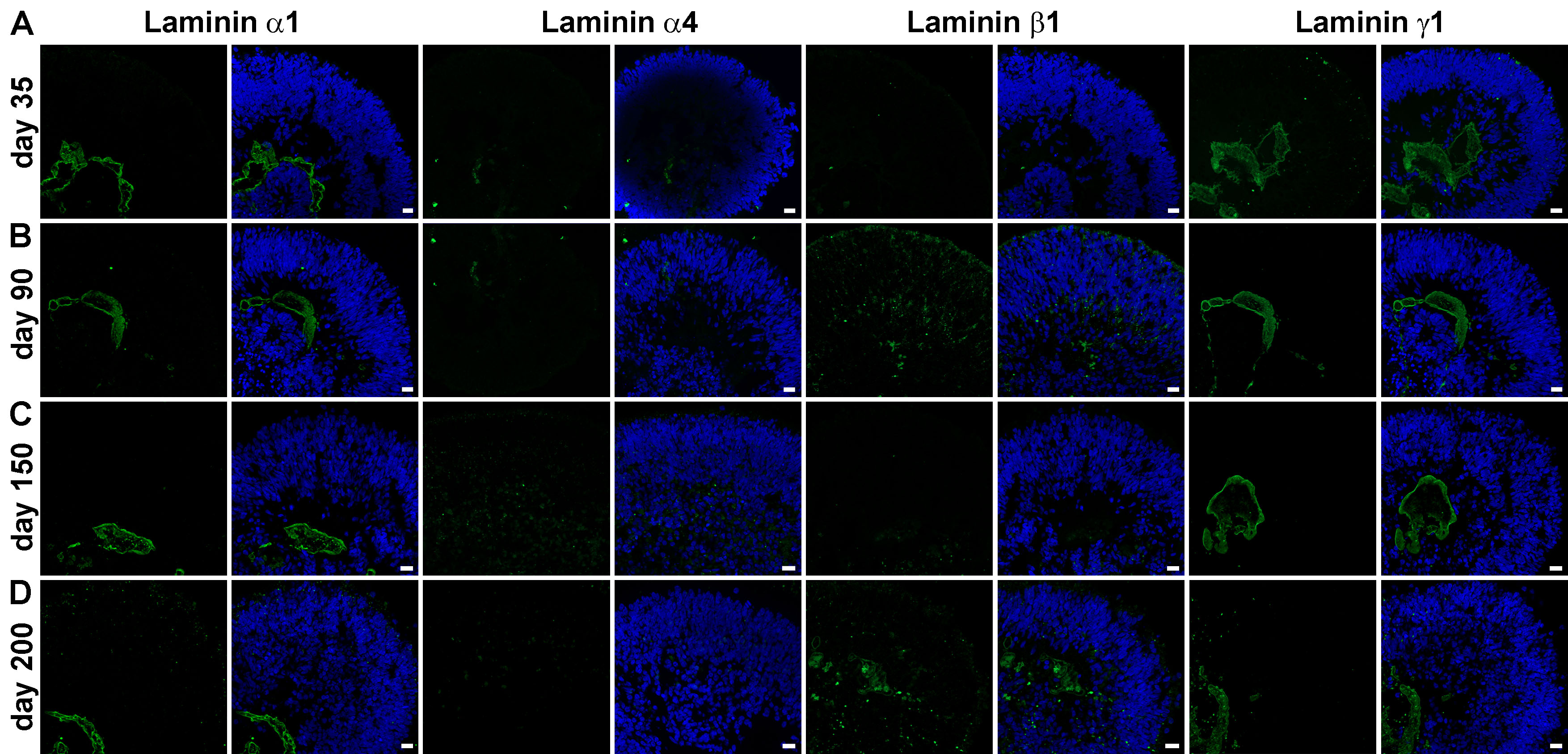

Supplement: Supplementary file 5 — Figure S4 [file 41419_2018_648_MOESM5_ESM.jpg]

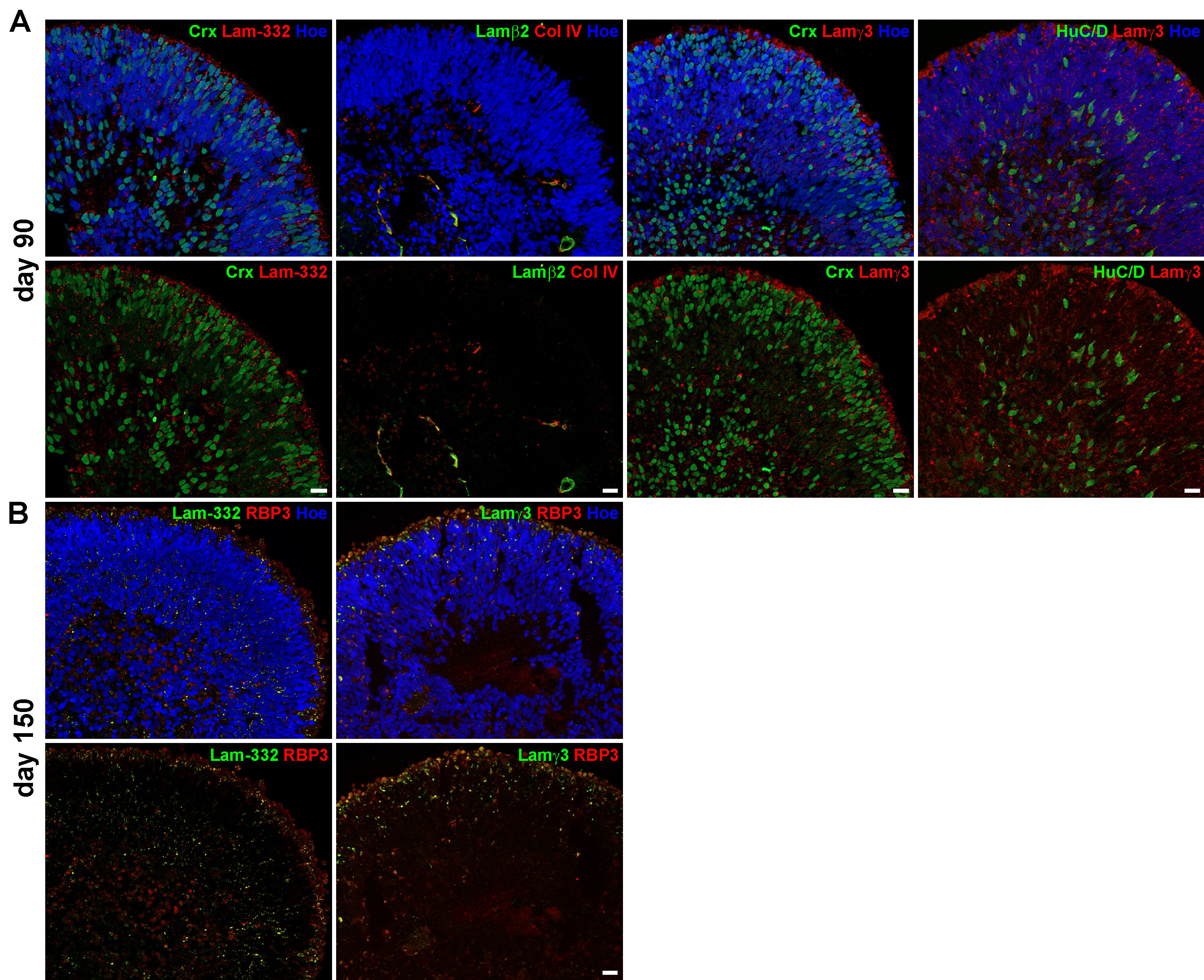

Supplement: Supplementary file 6 — Figure S5 [file 41419_2018_648_MOESM6_ESM.jpg]

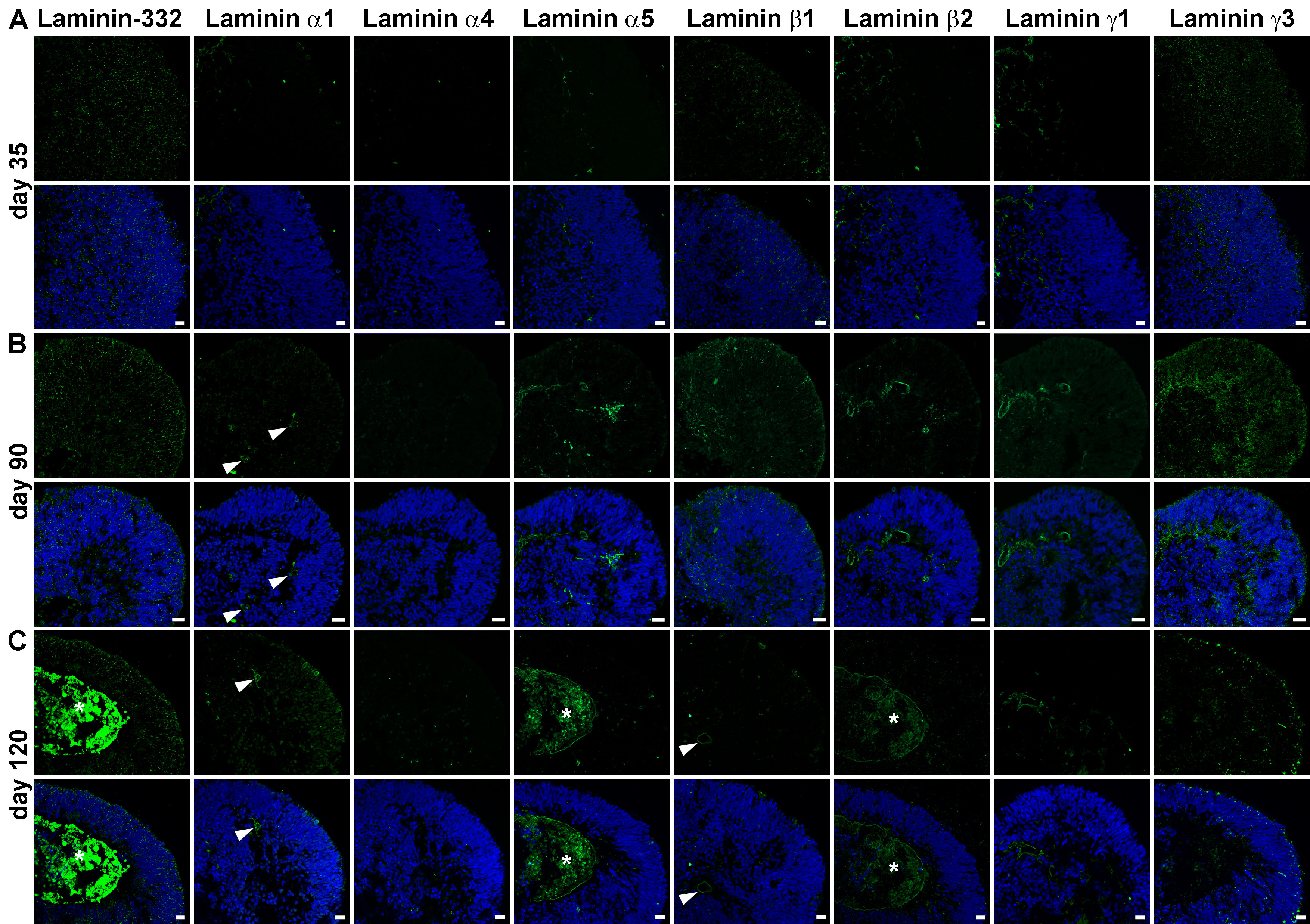

Supplement: Supplementary file 7 — Figure S6 [file 41419_2018_648_MOESM7_ESM.jpg]

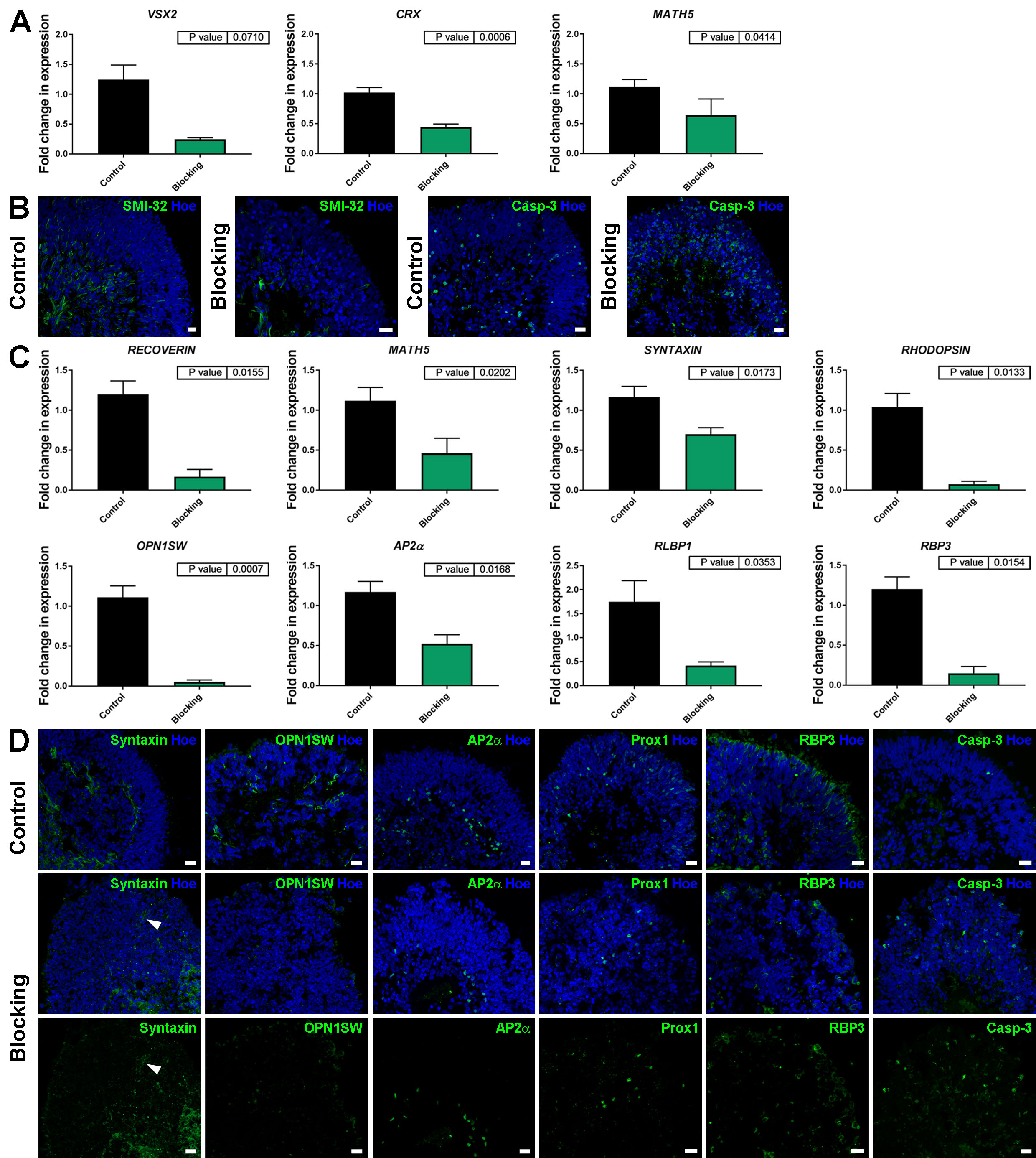

Supplement: Supplementary file 8 — Figure S7 [file 41419_2018_648_MOESM8_ESM.jpg]
