# Supplementary material for: Laminin γ3 plays an important role in retinal lamination, photoreceptor organisation and ganglion cell differentiation
Source: Cell Death Dis. 2018 May 23;9(6):615. doi: 10.1038/s41419-018-0648-0 (PMC5966411; doi:10.1038/s41419-018-0648-0)
Supplement: Supplementary file 9 — List of primary antibodies used for immunohistochemistry [file 41419_2018_648_MOESM9_ESM.docx]

**Supplement Table 1:** List of primary antibodies used for immunohistochemistry

| **Antibody** | **Immunogen** | **Host and clonality** | **Dilution** | **Supplier, Cat. No** |
| --- | --- | --- | --- | --- |
| AP2α, clone 3B5 | N-terminus of AP-2α of human origin | Mouse, monoclonal | 1:100 | Santa Cruz Biotechnology Inc., sc-12726 |
| Basson, clone SAP7F407 | Recombinant rat Bassoon | Mouse, monoclonal | 1:100 | Enzo Life Sciences LTD, ADI-VAM-PS003 |
| Cleaved Casapae-3, Asp175 | Synthetic peptide corresponding to amino-terminal residues adjacent to (Asp175) in human caspase-3 | Rabbit, polyclonal | 1:300 | Cell Signaling Technology,  9661S |
| CRX, clone 4G11 | CRX partial recombinant protein with GST tag | Mouse, monoclonal | 1:200 | Abnova, H00001406-M02 |
| CRALBP, clone B2 | Human recombinant CRALBP | Mouse, monoclonal | 1:100 | GeneTex, GTX15051 |
| HuC/HuD, clone 16A11 | Human HuC/HuD neuronal protein | Mouse, monoclonal | 1:200 | Invitrogen, A21271 |
| Laminin 5 | Full length native protein (purified) corresponding to Human Laminin 5 | Rabbit, polyclonal | 1:100 | Abcam, ab14509 |
| Laminin α1 | synthetic peptide from  human LAMA1 (aa2501-2550). | Rabbit, polyclonal | 1:200 | LifeSpan BioScience, Inc., LS-C119557 |
| Laminin α4, clone 3H2 | Recombinant protein corresponding to human Laminin subunit alpha-4 | Mouse, monoclonal | 1:200 | Millipore,  MABT38 |
| Laminin α5, clone 4C7 | Purified human laminin | Mouse, monoclonal | 1:100 | Millipore, MAB1924 |
| Laminin β1, clone 4E10 | Purified human laminin | Mouse, monoclonal | 1:100 | Millipore,  MAB1921 |
| Laminin β2, clone 5A | Murine EHS laminin | Rat, monoclonal | 1:100 | Millipore, 05-206 |
| Laminin γ1, clone B17 | raised against Human LAMC1 | Mouse, monoclonal | 1:100 | LifeSpan BioScience, Inc., LS-C202513 |
| Laminin γ3 | Synthetic peptide from human LAMC3 (aa1361-1410) | Rabbit, polyclonal | 1:100 | LifeSpan BioScience, Inc., LS-C119566 |
| Opsin, clone RET-P1 | Rat retinal membranes | Mouse, monoclonal | 1:200 | Sigma-Aldrich, O4886 |
| Opsin red/green | Recombinant human red/green opsin | Rabbit, polyclonal | 1:200 | Millipore, ab5405 |
| Opsin blue | Recombinant human blue opsin | Rabbit, polyclonal | 1:200 | Millipore, ab5407 |
| Pan Laminin | laminin purified from the basement membrane of Englebreth Holm-Swarm (EHS) mouse sarcoma | Rabbit, polyclonal | 1:200 | Sigma-Aldrich, L9393 |
| Prox1 | Synthetic peptide from the C-terminus of mouse Prox1 | Rabbit, polyclonal | 1:1500 | Millipore, ab5475 |
| Recoverin | Recombinant human recoverin | Rabbit, polyclonal | 1:1000 | Millipore, ab5585 |
| RBP3 | RBP3 fusion protein Ag5699 | Rabbit, polyclonal | 1:200 | ProteinTech, 14352-1-AP |
| SMI-32 | whole rat hypothalamus homogenate | Mouse, monoclonal | 1:200 | Covance, SMI-32P |
| Syntaxin, clone HPC-1 | synaptosomal plasma-membrane fraction from adult rat hippocampus | Mouse, monoclonal | 1:200 | Sigma-Aldrich, S0664 |
| vGlut1 | Synthetic peptide from rat VGLUT1 protein with no overlap to VGLUT2 | Guinea pig, polyclonal | 1:1500 | Millipore, AB5905 |
| VSX2 | Visual system homeobox 2 recombinant protein epitope signature tag (PrEST) | Rabbit, polyclonal | 1:50 | Sigma-Aldrich, HPA003436 |
| ZO-1 | 69 kD fusion protein corresponding to amino acids 463-1109 of human ZO-1 cDNA | Rabbit, polyclonal | 1:200 | Invitrogen, 61-7300 |
