# Supplementary material for: Laminin γ3 plays an important role in retinal lamination, photoreceptor organisation and ganglion cell differentiation
Source: Cell Death Dis. 2018 May 23;9(6):615. doi: 10.1038/s41419-018-0648-0 (PMC5966411; doi:10.1038/s41419-018-0648-0)
Supplement: Supplementary file 10 — List of primers used for qRT-PCR [file 41419_2018_648_MOESM10_ESM.docx]

**Supplement Table 2:** List of primers used for qRT-PCR

| **Gene** | | **Primer sequence** |
| --- | --- | --- |
| *AP2α* | *Fwd* | GTTACCCTGCTCACATCACTAG |
|  | *Rev* | TCTTGTCACTTGCTCATTGGG |
| *CRX* | *Fwd* | GTGAGGAGGTGGCTCTGAAG |
|  | *Rev* | CTGCTGTTTCTGCTGCTGTC |
| *GAPDH* | *Fwd* | TGCACCACCAACTGCTTAGC |
|  | *Rev* | GGCATGGACTGTGGTCATGAG |
| *MATH5* | *Fwd* | CCCTAAATTTGGGCAAGTGAAGA |
|  | *Rev* | CAAAGCAACTCACGTGCAATC |
| *OPN1SW* | *Fwd* | ATACCGCAGCGAGTCCTATAC |
|  | *Rev* | GATCCTACCATCACAACCAC |
| *RBP3* | *Fwd* | AGATCATGCACACGGATGCC |
|  | *Rev* | AGCCATAGCGTTCACCTACA |
| *RECOVERIN* | *Fwd* | TTCAAGGAGTACGTCATCGCC |
|  | *Rev* | GATGGTCCCGTTACCGTCC |
| *RHODOPSIN* | *Fwd* | TTTGGAGGGCTTCTTTGCCA |
|  | *Rev* | CCTCGGGGATGTACCTGGAC |
| *RLBP1* | *Fwd* | GGCAGGGAACAACCAAGACT |
|  | *Rev* | AGTCAGGGCCAAGTTGTGAC |
| *Syntaxin* | *Fwd* | AGAGCCAGGGAGAGATGATTGA |
|  | *Rev* | GGTGGCTTCTAGGCGAAGAT |
| *VSX2 (CHX10)* | *Fwd* | GGCGACACAGGACAATCTTTA |
|  | *Rev* | TTCCGGCAGCTCCGTTTTC |
