# Supplementary material for: Laminin γ3 plays an important role in retinal lamination, photoreceptor organisation and ganglion cell differentiation
Source: Cell Death Dis. 2018 May 23;9(6):615. doi: 10.1038/s41419-018-0648-0 (PMC5966411; doi:10.1038/s41419-018-0648-0)
Supplement: Supplementary file 11 — Laminin expression in adult mouse, macaque and human retina [file 41419_2018_648_MOESM11_ESM.doc]

**Supplement Table 3:** Laminin expression in adult mouse, macaque and human retina

|  | **Mouse** | **Macaque** | **Human** |
| --- | --- | --- | --- |
| **Laminin-332** | BrM, ILM | IPM, OPL, GCL | ONL, INL, GCL |
| **Laminin α1** | No | No | No |
| **Laminin α4** | BrM | BrM, ILM | No |
| **Laminin α5** | BrM | No | No |
| **Laminin β1** | BrM | No | No |
| **Laminin β2** | BrM | No | No |
| **Laminin γ1** | BrM | BrM | ILM |
| **Laminin γ3** | INL, GCL | IPM, INL, IPL | ONL, INL, GCL |

BrM, Bruch’s membrane; IPM, interphotoreceptor matrix; ONL, outer nuclear layer; OPL, outer plexiform layer; INL, inner nuclear layer; IPL, inner plexiform layer; GCL, ganglion cell layer; ILM, inner limiting membrane
