# Supplementary material for: Laminin γ3 plays an important role in retinal lamination, photoreceptor organisation and ganglion cell differentiation
Source: Cell Death Dis. 2018 May 23;9(6):615. doi: 10.1038/s41419-018-0648-0 (PMC5966411; doi:10.1038/s41419-018-0648-0)
Supplement: Supplementary file 12 — Laminin expression in human developing eye [file 41419_2018_648_MOESM12_ESM.doc]

**Supplement Table 4:** Laminin expression in human developing eye

|  | **6.3 PCW** | **8 PCW** | **10 PCW** | **12 PCW** | **14 PCW** | **16 PCW** | **18 PCW** |
| --- | --- | --- | --- | --- | --- | --- | --- |
| **Laminin-332** | BrM, Retina | BrM, Retina | BrM, IPL,  INBZ | ONBZ, IPL, INBZ | BrM, IPM, ONBZ, IPL, INBZ | BrM, IPM, ONBZ, IPL, INBZ | BrM, IPM, ONBZ, IPL, INBZ |
| **Laminin α1** | BrM, ILM | No | BrM | ILM | No | ILM | ILM |
| **Laminin α4** | No | No | No | BrM, IPM, ILM | IPM, ONBZ | No | No |
| **Laminin α5** | BrM, ILM | BrM, ILM | BrM, ILM | BrM, ONBZ, INBZ, IPL, ILM | BrM, ILM | BrM, ILM | BrM, ILM |
| **Laminin β1** | No | No | No | BrM, ILM | BrM | No | BrM, ILM |
| **Laminin β2** | BrM, ILM | BrM, ILM | BrM, ILM | BrM, ILM | BrM, ILM | BrM, INBZ, ILM | BrM, ILM |
| **Laminin γ1** | Retina, ILM | BrM | ILM | BrM, ILM | BrM | BrM, ILM | BrM, ILM |
| **Laminin γ3** | Retina | Retina | IPM, IPL, INBZ | IPM, IPL, INBZ | IPM, ONBZ, IPL, INBZ | IPM, ONBZ, IPL, INBZ | IPM, ONBL, IPL, GCL |

BrM, Bruch’s membrane; RPE, retinal pigment epithelium; IPM, interphotoreceptor matrix; ONBZ, outer neuroblastic zone; INBZ, inner neuroblastic zone; INL, inner nuclear layer; IPL, inner plexiform layer; GCL, ganglion cell layer; ILM, inner limiting membrane; PCW, post conceptual week
